# Reduction of APOE accounts for neurobehavioral deficits in fetal alcohol spectrum disorders

**DOI:** 10.1038/s41380-024-02586-6

**Published:** 2024-05-11

**Authors:** Hye M. Hwang, Satoshi Yamashita, Yu Matsumoto, Mariko Ito, Alex Edwards, Junko Sasaki, Dipankar J. Dutta, Shahid Mohammad, Chiho Yamashita, Leah Wetherill, Tae-Hwi Schwantes-An, Marco Abreu, Amanda H. Mahnke, Sarah N. Mattson, Tatiana Foroud, Rajesh C. Miranda, Christina Chambers, Masaaki Torii, Kazue Hashimoto-Torii

**Affiliations:** 1 Center for Neuroscience Research, The Children’s Research Institute, Children’s National Hospital, Washington, DC USA; 2 Department of Diabetes, Endocrinology and Metabolism, Tokyo Medical University, Tokyo, Japan; 3 Department of Medical and Molecular Genetics, Indiana University School of Medicine, Indianapolis, IN USA; 4 Department of Neuroscience and Experimental Therapeutics, Texas A&M University School of Medicine, Bryan, TX USA; 5 Center for Behavioral Teratology, San Diego State University, San Diego, CA USA; 6 Department of Pediatrics, University of California San Diego, San Diego, CA USA; 7 Departments of Pediatrics, and Pharmacology & Physiology, School of Medicine and Health Sciences, The George Washington University, Washington, DC USA

**Keywords:** Neuroscience, Biomarkers, Diseases

## Abstract

A hallmark of fetal alcohol spectrum disorders (FASD) is neurobehavioral deficits that still do not have effective treatment. Here, we present that reduction of Apolipoprotein E (APOE) is critically involved in neurobehavioral deficits in FASD. We show that prenatal alcohol exposure (PAE) changes chromatin accessibility of *Apoe* locus, and causes reduction of APOE levels in both the brain and peripheral blood in postnatal mice. Of note, postnatal administration of an APOE receptor agonist (APOE-RA) mitigates motor learning deficits and anxiety in those mice. Several molecular and electrophysiological properties essential for learning, which are altered by PAE, are restored by APOE-RA. Our human genome-wide association study further reveals that the interaction of PAE and a single nucleotide polymorphism in the *APOE* enhancer which chromatin is closed by PAE in mice is associated with lower scores in the delayed matching-to-sample task in children. APOE in the plasma is also reduced in PAE children, and the reduced level is associated with their lower cognitive performance. These findings suggest that controlling the APOE level can serve as an effective treatment for neurobehavioral deficits in FASD.

## Introduction

Fetal alcohol spectrum disorders (FASD) is an umbrella term for neurodevelopmental disorders caused by prenatal alcohol exposure (PAE). The clinical presentation of FASD is heterogenous, including intellectual disability, delay in motor and language development, and other psychiatric and neurological problems [1]. According to the Centers for Disease Control and Prevention, about 10% of women consume alcohol while pregnant in the US, suggesting that the estimated prevalence of FASD in 1–5% (weighted estimate of 3.1–9.85%) in school-aged children is conservative [2]. Studies have suggested that the manifestations of FASD are due to multifaceted changes caused by the epigenetic effect of alcohol [3]. While PAE causes FASD, not all exposures result in the same FASD outcomes. For instance, only 4.3% of children with heavy exposure to ethanol develop fetal alcohol syndrome (FAS), the most severe form of FASD [4, 5]. Furthermore, twin studies revealed that fraternal twins have vastly different FASD outcomes whereas identical twins exhibit similar FASD outcomes [6, 7]. This evidence suggests that genetic factors modify fetal susceptibility to PAE. However, a lack of understanding of predisposing genetic factors that interact with alcohol exposure and a lack of knowledge about epigenetic effects of PAE have served as an impediment to developing effective treatment for neurocognitive problems in FASD.

This study reports potential mechanism of treatment for patients with PAE could involve the gene that encodes the protein called apolipoprotein E (*APOE*). *APOE* controls synaptic plasticity, which is crucial for brain function [8]. For instance, *Apoe*-deficient mice show a decreased number of synapses in the brain, including the frontal cortex and hippocampus, without obvious gross structural abnormalities [9–11]. *APOE* receptors such as low-density lipoprotein receptor related protein 1 (*LRP1*), are localized at the postsynaptic membrane, and abolishing these receptors in neurons results in impaired motor function [12]. However, the impact of PAE on APOE function and the involvement of APOE in the pathogenesis of FASD have not been explored.

In this study, we identified an epigenetic mechanism that drives the reduction in brain APOE in PAE mice. Our data also showed that postnatal administration of an APOE receptor agonist (APOE-RA) rescued both learning deficits and anxiety in PAE mice. In humans, we found that a single nucleotide polymorphism (SNP) within an *APOE* enhancer, in the presence of PAE, is associated with decreased cognitive measures compared to individuals with the same genotype without PAE. Finally, APOE level was reduced in the plasma of PAE children, correlated with their lower cognitive performance. These results indicate that reduced APOE expression due to PAE critically contributes to neurocognitive deficits, and a functional *APOE* polymorphism is a genetic risk factor that augments the effects of PAE on cognitive performance. Improvement of learning deficits when APOE was increased via an agonist in PAE mice suggest that APOE replenishment is a potential therapy for FASD patients.

## Materials and methods

### Animal model

Timed pregnant CD-1 mice were purchased from Charles River Laboratories and maintained on a light-dark cycle (lights on 6:00–18:00) at a constant temperature (22 ± 1 °C). Pregnant mice were randomly assigned to experimental groups. To generate PAE mice [13, 14], we administered a single dose of 4 g/kg body weight ethanol to pregnant mice via intraperitoneal (i.p.) injection at E16 and E17. Same amount of PBS was administered to generate control mice. Experiments were conducted at around P30, unless otherwise noted. All studies employed a mixture of males and females, and no differences between sexes were observed. All protocols were approved by the Institutional Animal Care and Use Committee (IACUC) of Children’s National Hospital. The number of animals and statistical parameters are denoted in the figure legends.

### Accelerated rotarod test

An accelerated rotarod test was performed as described previously [13]. Briefly, each mouse was placed on a rotating bar, and the time the mouse could maintain balance while the rotation is accelerated to the maximum speed (80 RPM) in 5 min was measured. The testing phase consisted of 2 consecutive days of three trials per day. Each trial was at least 15 min apart and was terminated when the mouse fell off, made one complete rotation without walking on the rotating rod, or reached the maximum speed after the 5-min session. The latency to fall from the rotating rod was scored by automatic timers and falling sensors on the rotarod. Learning index was calculated by averaging the changes in terminal speed of an individual mouse between two consecutive trials.

### Elevated plus maze test

The maze is a grey plus-shaped apparatus with two open arms and two closed arms linked by a central platform. Mice were individually put in the center of the maze facing an open arm and allowed to explore the maze for 5 min. A video was recorded during the experiment, and the time spent in open and closed arms was measured and analyzed with MoutBeat ImageJ Plugin as previously done [14].

### Isolation of peripheral blood mononuclear cells

Mice were deeply anesthetized with isoflurane (Henry Schein, NY, US). Blood was then drawn from their heart via cardiac puncture, immediately transferred to collection tubes containing EDTA-2K, and centrifuged at 1500 RPM for 5 min at room temperature. The top layer, containing primarily blood plasma, was discarded. The remnant, containing peripheral blood mononuclear cells (PBMCs), was mixed with 5 ml of 10% FBS in DMEM/F12 (cat# 11320033, Thermo Fisher Scientific, MA, US) and Ficoll-Paque PLUS solution (cat# GE17-1440-02, Millipore Sigma, MA, US) and centrifuged at 1000 × *g* for 15 min at room temperature. The middle layer containing PBMCs was collected, washed twice with PBS, and then transferred to ice-cold FACS (Fluorescence-activated cell sorting) buffer for FACS analysis.

### Fluorescence-activated cell sorting (FACS)

PBMCs were stained with rat monoclonal anti-mouse CD11b antibody (FITC conjugated, cat# 101205, Biolegend, CA, US), CD19 antibody (PE-conjugated, cat# 152407, Biolegend), and CD90.2 antibody (APC conjugated, cat# 105311, Biolegend). Cells were first gated based on cell size [forward scatter (FSC) versus side scatter (SSC)] and singlets (FSC versus trigger pulse width). Then T cells, B cells, and monocytes were separated from each other by collecting the CD11b+/CD19−, CD19+/CD90.2−, and CD19−/CD90.2+ subpopulations of cells, respectively.

### Library preparation and RNA sequencing

The cDNA libraries were prepared using the SMART-Seq v4 Ultra Low Input RNA Kit for Sequencing (cat# 634888, Takara Bio, Kusatsu, Japan) and Nextera XT DNA Library Prep Kit (cat# FC-131-1096, Illumina, CA, US) as per manufacturer instructions. The unique barcode sequences were incorporated in the adaptors for multiplexed high-throughput sequencing. The final product was assessed for its size distribution and concentration using Bioanalyzer High Sensitivity DNA Kit (cat# 5067-4626, Agilent, CA, USA). The libraries were pooled and diluted to 3 nM with 10 mM Tris-HCl, pH 8.5, then denatured using the Illumina protocol. According to manufacturer instructions, the denatured libraries were loaded onto an S1 flow cell on an Illumina NovaSeq 6000 (Illumina) and ran for 2 × 50 cycles. De-multiplexed sequencing reads were generated using Illumina bcl2fastq (released version 2.18.0.12), allowing no mismatches in the index read.

### RNA-seq data analysis of PBMC transcriptome

Low quality reads from the RNA-seq dataset were removed via FastQC (sickle with default setting; available at https://www.bioinformatics.babraham.ac.uk/projects/fastqc/). A HISAT2 index was built for the mm10 genome assembly using HISAT2 v2.1.0 [15]. RNA-sequencing reads of each sample were mapped using HISAT2 or STAR v2.5.4a supplied [16] with Ensembl annotation file; GRCm38.78.gtf. For the count call, HTseq-count v0.10.0 [17] or featurecounts [18] was used. Differential gene expression analysis of count data was performed using IRIS Web server-based (http://bmbl.sdstate.edu/IRIS/) limma-voom v3.38.3 [19]. First, raw counts were filtered by a cutoff value of count data row sums less than 10 and transformed to log2 (n + pseudocount). Then, limma-voom was carried out with a minimum fold change of 2 and an adjusted *p*-value cutoff of 0.05. A volcano plot was generated with the Galaxy platform (https://usegalaxy.eu/). Quantile normalized counts of differentially expressed genes were clustered by hierarchical k-means clustering using Morpheus (https://software.broadinstitute.org/morpheus/). The optimal number of clusters was determined by the within sum scale (WSS) method with “wssplot” function in R. Gene ontology enrichment analysis was performed suing Enrichr. The combined scores were generated by taking the log of the *p*-value from the Fisher exact test and multiplied that by the z-score of the deviation from the expected rank by Enrichr. For Ingenuity Pathway Analysis (IPA; Qiagen, Venlo, Netherlands), cutoff values of expression log ratio of ±1.5 and FDR < 0.05 or 0.025 were used to limit the number of genes to be less than 2000 as recommended per the company.

### Quantification of plasma APOE and corticosterone in mice

Whole blood was collected in an EDTA-treated tube (cat# 365974, BD, NJ, USA) by cardiac puncture with a 23-25 G needle from a deeply anesthetized animal. The plasma was separated from blood cells by centrifuging the blood sample at 2000 × *g* at 4 °C for 10 min and transfering the supernatant to a clean centrifuge tube. Collected plasma samples were stored in a –80 °C freezer until further analysis. Measurements of APOE and corticosterone were carried out using ELISA Pro: Mouse apoE (cat# 3752-1HP-1, Mabtech, Nacka Strand, Sweden) and Mouse Corticosterone ELISA Kit (cat # 80556, Crystal Chem, IL, USA), respectively following manufacturer’s protocols.

### Free-floating immunohistochemistry

Mice were deeply anesthetized with isoflurane (Henry Schein) and perfused transcardially with 10 ml of ice-cold PBS followed by 10 ml of chilled 4% paraformaldehyde (PFA). The brains were removed and post-fixed in the same fixative (4% PFA) at 4 °C overnight. The brains were then incubated in 10% and 30% sucrose solution prepared in PBS for 24 h sequentially at 4 °C. After embedding in OCT, coronal sections were cut at 20 or 50 µm on a cryostat (CM3050S, Leica, Wetzlar, Germany).

Immunohistochemstry was performed on free-floating brain sections as follows: Antigen retrieval was performed when necessary following the manufacturer’s protocol (cat# 00-4955-58, Thermo Fisher Scientific). Then sections were incubated in 30% hydrogen peroxide in methanol (1:4) solution for 30 min at −20 °C to inactivate endogenous peroxidase activity. After rinsing with PBS-T (0.1% Tween-20 in PBS), sections were incubated with 2% BSA for 30 minutes at room temperature for blocking. Sections were then incubated with a primary antibody for APOE (1:500, cat# ab1906, Abcam, Cambridge, UK), NeuN (1:500, cat# ab104225, Abcam), GFAP (1:500, cat# ab4674, Abcam), GluN1 (1:200, cat# 32-0500, Thermo Fisher Scientific), LRP1(1:200, cat# ab92544, Abcam), PSD-95 (1:50, cat# MAI-045, Thermo Fisher Scientific) or KCNN2 (1:500, cat# HPA038221, Sigma-Aldrich, MO, USA) overnight at 4 °C. The next day, after washing with PBS-T, sections were incubated with a secondary antibody: horseradish peroxidase (HRP)-conjugated anti-mouse IgG (cat# 111-035-146, Jackson ImmunoResearch, PA, US) or anti-rabbit IgG (cat# 111-035-144, Jackson ImmunoResearch), or biotinylated anti-rabbit IgG (cat# 711-065-152, Jackson ImmunoResearch), diluted at 1:300 for 2 h at room temperature. After washing with PBS-T, sections incubated in the HRP-conjugated secondary antibody were incubated in TSA solution (1:500 diluted in TSA diluent) for 1 h at room temperature. Sections incubated with the biotinylated secondary antibody were incubated in avidin-biotin complex (ABC; cat# 32020, Thermo Fisher Scientific) solution for 1 h, followed by 1-h incubation in TSA solution (1:500 diluted in TSA diluent) at room temperature. The following TSA fluorophores were used: TSA plus Fluorescein (cat# NEL741001KT, Akoya Biosciences, MA, USA), Cyanine-3 (cat# NEL744001KT, Akoya Biosciences), Cyanine-5 (cat# NEL745001KT, Akoya Biosciences). For NeuN and GFAP staining, Alexa Fluor 488-conjugated secondary antibody (cat# 703-545-155, Jackson ImmunoResearch) was used at 1:300 dilution for 1 h incubation. After washing with PBS-T, sections were counterstained with DAPI and mounted using CC/Mount aqueous mounting medium (cat# C9369, Sigma-Aldrich).

### Chemicals

COG-133 trifluoroacetate salt (APOE-RA) was purchased from Sigma-Aldrich (cat# C8624) and dissolved in PBS for i.p. injections (1 mg/kg body weight). The dosage and duration of treatment were determined based on previous studies using the same APOE mimetic peptide in other disease models [20, 21]. To determine the localization of COG-133 after i.p. injection, N-terminus biotinylated COG-133 was synthesized by GenScript. Mouse brains were collected 5 min after the injection of biotinylated COG-133 and fixed with 4% PFA. To detect biotinylated COG-133 bound in the brain, 50 µm cryosectioned brain slices were incubated with HRP-conjugated streptavidin (1:1000, cat# NEL750001EA, Akoya Biosciences) followed by TSA plus Cyanine-5 to stain for biotin. To also label LRP1 by immunohistochemistry (as described above), slices were incubated in anti-LRP1 primary antibody (1:200, cat# ab92544, Abcam) followed by HRP-conjugated secondary antibody and TSA plus Fluorescein (1:500 in TSA diluent buffer).

### Blood-brain barrier permeability test

Sodium fluorescein (100 µl of 100 mg/ml; cat# F6377, Sigma-Aldrich) or Evans blue (200 µl of 20 mg/ml; cat# E2129, Sigma-Aldrich) was administered via i.p. injection to control and PAE mice. Tracers were allowed to circulate the body for 30 minutes, and brains were perfused as described above. Brains were sectioned with a microtome (Leica VT1000 S) at 100 µm and imaged with a BX63 microscope.

### Electrophysiology

Coronal brain slices (300 µm) at the level of the motor cortex were prepared from P30-40 mice using a vibratome (VT 1200S) as described previously [13, 22]. Briefly, brain slices were transferred to a submerged recovery chamber with oxygenated (95% O_2_ and 5% CO_2_) artificial cerebrospinal fluid (ACSF) solution containing 125 mM NaCl, 2.5 mM KCl, 2 mM CaCl_2_, 2 mM MgCl_2_, 25 mM NaHCO_3_, 1 mM NaH_2_PO_4_–H_2_O, and 25 mM glucose at room temperature for at least 1 h. Individual slices were then put into a recording chamber in which bath ACSF solution was continuously perfused at 32–33 °C. Bath ACSF solution was obtained by omitting MgCl_2_ from the ACSF solution. Patch-clamp recordings were made from single neurons visualized with infrared differential interference contrast optics (BX51WI, Olympus, Tokyo, Japan). Whole-cell patch-clamp recordings were made from layer V pyramidal neurons in the motor cortex. Pyramidal neurons were identified based on their large size and teardrop morphology. The patch pipettes (2–5 MΩ) were filled with a cesium-based intracellular solution containing 130 mM Cs^+^ -gluconate, 10 mM CsCl, 1 mM CaCl_2_, 11 mM EGTA, 2 mM ATP-Mg, and 10 mM HEPES (pH 7.2) for recordings of excitatory postsynaptic currents (EPSCs). sNMDA EPSCs were recorded under voltage-clamp conditions at a holding potential of +50 mV in the presence of 10 µM bicuculline, 5 µM strychnine, and 10 µM 1,2,3,4-Tetrahydro-6-nitro-2,3-dioxo-benzo[f]quinoxaline-7-sulfonamide (NBQX). The recording signals were amplified with a patch-clamp amplifier (MultiClamp 700B; Molecular Devices, CA, USA), low-pass filtered at 5 kHz, digitized with an analog-to-digital converter (Digidata 1440 A; Molecular Devices), and stored on a personal computer using a data acquisition program (Clampex 10.7; Molecular Devices) with a sampling frequency of 10 kHz. Synaptic events were analyzed offline with Clampfit 10.7 software (pCLAMP10, Molecular Devices). Peak, 10–90% rise times, and decay time constants (Tau) of EPSCs were calculated using standard algorithms. Only recordings with the access resistance <20 MΩ and less than 20% change throughout the experiment were included for analysis.

### Immunoblotting of CREB

Motor cortex, cerebellum, and striatum tissues were dissected from mice and frozen immediately in liquid nitrogen and stored at –80 °C until use. Brain tissues were homogenated in ice-cold T-PER reagent (cat# 78510, Thermo Fisher Scientific) containing Halt protease and phosphatase inhibitor cocktail (cat# 78430, Thermo Fisher Scientific) and EDTA for 15 s on ice. Tissue lysates were subsequently centrifuged at 10,000 × *g* for 5 min at 4 °C. The soluble portion of the lysates was collected for analysis.

Each protein sample was separated on NuPAGE 4–12% Bis-Tris gel (cat# NP032, Thermo Fisher Scientific). The separated proteins were transferred onto a PVDF membrane, probed with respective primary antibodies, and exposed to an HRP-conjugated secondary antibody. The following primary antibodies were used: anti-CREB (48H2) rabbit monoclonal antibody (cat# 9197, Cell Signaling Technology, MA, CA), and anti-phospho-CREB (Ser-133) rabbit monoclonal antibody (cat# 9198, Cell Signaling Technology). The reactive protein bands were visualized by chemiluminescence with the Amersham Imager 680 (GE Healthcare, IL, US) using the Super Signal West Pico Plus Chemiluminescent Substrate (Thermo Fischer Scientific). Subsequently, membranes were stripped with Restore Western Blot Stripping Buffer (cat# 21059, Thermo Fisher Scientific) and reprobed with the mouse monoclonal antibody against GAPDH (cat# sc-47724, Santa Cruz, CA, US), followed by the same procedure to detect the reactive protein bands. Quantification of bands was done by densitometry using FIJI/Image J. For the analysis of CREB phosphorylation, the optical densities of phosphorylated CREB bands were measured relative to those of total CREB from the same brain sample. Statistical analysis was done with Student’s *t*-test, and the threshold of significance was defined as *P* < 0.05.

### RNAscope in situ hybridization

To detect mRNA molecules, RNAscope in situ hybridization was performed on PFA-fixed 20 µm-thick sections following the manufacturer’s protocol. *Apoe* probes (cat# 313271, Advanced Cell Diagnostics, CA, USA) were detected with TSA plus Fluorescein, followed by immunohistochemistry for NeuN by incubating the sections with an anti-NeuN primary antibody (1:200, cat# MAB377, EMD Millipore, MA, US), HRP-conjugated secondary antibody, and TSA plus Cyanine-5. *Grin2a* (cat# 481831, Advanced Cell Diagnostics) and *Grin2b* (cat# 417391-C2, Advanced Cell Diagnostics) probes were detected with TSA plus Fluorescein and TSA plus Cyanine-5, respectively. All samples were counterstained with DAPI before placing coverslips.

### Image acquisition and analysis

All images were taken by confocal microscopy (FV1000, Olympus). Allen Brain Atlas (https://mouse.brain-map.org/static/atlas) was used to define different brain regions visualized by DAPI staining. APOE, GFAP, GluN1, and LRP1-positive cells were quantified by first manually identifying DAPI-positive cells that are also immunolabeled, and then counting those immunolabeled cells within a region of interest using the cell counter plugin in ImageJ. To quantify the levels (total intensity) of APOE immunolabeling and biotinylated COG-133 detection per cell, the areas of individual cells were isolated and the staining intensity within each area was measured using Image J.

To count KCNN2 and PSD-95 puncta, automatic particle counting was performed using the particle analysis plugin in ImageJ. A colocalization tool in cellSens was used for colocalization analysis, and colocalization coefficients were reported.

Images of RNAscope in situ hybridization were analyzed following the method provided by the manufacturer. First, the total numbers of positive mRNA molecular signals and DAPI-positive cells were quantified separately using the automatic particle counting tool in ImageJ. Then the total number of mRNA puncta was divided by the total number of DAPI-positive cells to obtain the average number of mRNA molecules per cell.

### Generation of simulated datasets and comparison of effect sizes with the real data of APOE staining

To investigate the underlying causes of decreased APOE levels in (NeuN+) cortical neurons and (GFAP+) astrocytes in PAE mice—whether they are due to an overall decrease in APOE levels in all neurons/astrocytes (Uniform Reduction model) or loss of neurons/astrocytes with high levels of APOE (Selective Cell Loss model)—we juxtaposed the simulated dataset of APOE expression levels corresponding to each model against the actual data of PAE mice. The datasets corresponding to the Uniform Reduction model were created by multiplying the actual data of APOE levels in control mice by a fixed ratio so that the mean APOE level values of simulated data matched those of the actual data of PAE mice. The dataset corresponding to the Selective Cell Loss model was formulated by excluding the data points of top 16 neurons and the top 11 astrocytes with the highest APOE levels from the actual data of control mice, so that the mean APOE level values of these simulated datasets matched those of the actual data of PAE mice.

The formula used for calculation of Cohen’s d effect size is *d* = *M*_2_ *– M*_1_/*SD*_pooled_*. M*_1_ is the mean of the actual data of PAE mice or simulated data, and *M*_2_ is the mean of the actual data of control mice. *SD*_pooled_ is the pooled standard deviation.

### ATAC-seq

Cortical pyramidal neurons in the motor cortex were labeled with GFP that was introduced by *in utero* electroporation as described previously [13]. The pCAGIG plasmid was transfered into the motor cortex of E15 mouse embryos. Following electroporation, the pregnant mother received i.p. injection of ethanol at 4.0 g/kg weight or PBS daily at E16 and E17. At P15, the motor cortex was dissected and dissociated into single cells with Papain Dissociation System (cat# LK003150, Worthington, OH, US). The cells were FACsorted to collect GFP-positive cells. Dead cells were removed with SYTOX Red Dead Cell Stain (cat# S34859, Thermo Fisher Scientific). 500 cells in each group were tagmented with Illumina Tagment DNA Enzyme and Buffer Small Kit (cat# 20034197, Illumina).

For 500-cell-low-input ATAC-seq, a previously published protocol was utilized [23]. Lysis and transposition were conducted simultaneously with 10 μl of transposition mix (3.3 μl PBS, 1.15 μl water, 5 μl 2×TD Buffer, 0.25 μl Tn5 enzyme, 0.1 μl 1% digitonin, 0.1 μl 10% Tween-20, and 0.1 μl 10% NP40) at 37 °C for 30 min in a thermomixer shaking at 1000 RPM. Tagmented DNA was cleanup with a DNA Clean and Concentrator-5 kit (cat# D4004, Zymo Research, CA, USA) and amplified by PCR with index primers for 20 cycles. Purified libraries with Ampure XP (cat# A63880, Beckman Coulter, CA, USA) were sequenced with NovaSeq 6000 System (Illumina) as 2 ×50 bp paired-end readings.

The sequencing reads were aligned to the mouse reference genome (GRCm38/mm10) using Bowtie2 v2.3.4.3 [24] with parameters –very-sensitive -k 10. Mapped reads were filtered with Picard v2.25.0-0 (http://broadinstitute.github.io/picard/) for PCR duplicates, Samtools v1.12 [25] for mitochondrial DNA, and Bedtools v.2.30.0 [26] for ENCODE blacklist [27]. Multimapreads were removed using the grep command with the “XS” tag in SAM format. The distribution of aligned fragment length was analyzed with Qualimap v2.2.2 [28]. ATAC-seq peak regions were called using Genrich v0.6 (https://github.com/jsh58/Genrich) with parameters -j -y -r -v, and coverage tracks were drawn with Deeptools v3.5.0 [29] and SparK v2.6.2 [30], normalized by library size with count per million (CPM). Gene annotations were added with ChIPseeker v1.26.2 [31] in R v4.0.3.

### Genome-wide association study

Saliva was collected from participants across four sites (San Diego and Los Angeles, CA; Atlanta, GA; Minneapolis, MN) as part of the ongoing international consortium, the Collaborative Initiative on Fetal Alcohol Spectrum Disorders (CIFASD). All sites have approval from their individual Institutional Review Board. Informed consent was provided by all participants and/or their parents or legal guardians.

Genome-wide single nucleotide polymorphisms (SNPs) were genotyped by the Johns Hopkins SNP Center on the Illumina OmniExpress array (*n* = 234) or the Mega Consortium array (*n* = 313). Individual genotype and SNPs were cleaned according to standard procedures and then imputed using the Michigan Imputation Server [32] and the 1000 Genomes Reference panel. All SNP data are from genome build GRCh37/hg19. Exclusion criteria for individual SNPs were imputed information score <0.4, monomorphic SNPs, and SNPs with genotyping rates <99% across all genotyped samples. Principal components (PCs) estimating a continuum of allele frequency variation were calculated using SNPRelate [33]. Using 1000 Genome Project (1KGP) [34] as a reference population, we grouped the participants based on their genetic ancestry: African American, European American, and American Hispanics from Mexico, Puerto Rico, Columbia, and Peru. Participants from other genetic ancestry groups were too few to be analyzed and excluded from the analysis. SNPs included in analyses had a minor allele frequency (MAF) ≥ 0.01 and Hardy–Weinberg Equilibrium *p*-value ≥ 0.000001 within each genetic ancestry group.

Using an additive genetic model, PLINK 2 [35] was used to perform genome-wide association study (GWAS) analyses on the three ancestry groups separately. Covariates included sex, age at the time of the neuropsychological evaluation, the first 4 PCs, prenatal alcohol exposure (PAE), and the interaction of genotype*PAE. *P*-values for the genotype*PAE interaction were combined in a meta-analysis across the three ancestry groups using METAL [36]. To associate the phenotypes, we performed GWAS using the delayed matching to sample task (DMS) total z-score from the Cambridge Neuropsychological Test Automated Battery (CANTAB) using PLINK 2 with the covariates defined above. Significant (*p* < 0.05) SNPs associated with any of these three phenotypes across all three ancestry groups were examined. LocusZoom was used to visualize GWAS results near the *APOE* locus [37].

### APOE plasma test in Ukraine cohort

Plasma was collected from participants across two sites in Western Ukraine (Khmelnytsky Perinatal Center and Rivne Regional Medical Diagnostic Center) as part of the ongoing international consortium, the Collaborative Initiative on Fetal Alcohol Spectrum Disorders (CIFASD). Research and consent was approved by Institutional Review Boards at the University of California San Diego and Liviv Medical University in Ukraine. Informed consent was provided by all participants and/or their parents or legal guardians.

Pregnant women were recruited based on self-reported drinking behavior [38]. In the alcohol consuming group, women reported drinking either during the month of conception or the most recent month of pregnancy at least: weekly binge drinking (5+ drinks; five instances of 3–4 drinks; or 10 instances of 1–2 standard drinks). In the nonconsuming group, women reported no binge drinking, minimal to no drinking at conception, and no drinking in the most recent month of pregnancy.

Children were assessed at 6 and 12 months using the Bayley Scales of Infant Development (2nd edition) with standard scores for Mental Development Index (MDI), Psychomotor Development Index (PDI). Whole blood was collected in an EDTA-treated tubes Samples were centrifuged and plasma was aspirated, aliquoted, and stored in a –80 °C freezer. Samples were shipped on dry ice and store at –80 °C until analysis. Measurements of APOE (cat# 3712-*1HP*-*2*, Mabtech) were carried out following the manufacturer’s protocols.

We excluded children whose mothers have a smoking history, obesity before pregnancy (BMI greater than or equal to 30), or with low birth weight (birth weight less than 2800 × *g*) from analysis. Analysis was performed using SPSS software (SPSS version 28.0). We employed inverse probability weighting (IPW) to reduce the selection bias. We used 4 variables from maternal characteristics (age, socioeconomic index score, BMI before pregnancy, and recruitment site) and 4 variables from child characteristics (age, birth weight, sex, and developmental delay). Propensity scores were calculated with the logistic regression to predict the probability of each mother consuming alcohol. The propensity score-based IPW balanced the baseline characteristics between alcohol exposed and non-exposed groups.

### Statistical analysis

For all in vivo experiments, mice were excluded when we found health concerns, such as infection, bleeding, or significant body weight changes. All of the statistical analysis was carried out with GraphPad Prism 7.01. We performed the D’Agostino–Pearson to check for data normality. For the data that passed the normality test, two-tailed Student’s t-tests or one-way or two-way ANOVAs were used and followed by a Tukey’s or Bonferroni’s post hoc test as described in the figure legends. Simple main effects were reported when there was a statistically significant interaction between independent variables by two-way ANOVA. The two-tailed Mann–Whitney U test was used for two-group comparisons of data that did not follow a normal distribution. Pearson’s or Spearman’s correlation coefficient calculation was done for normally or nonnormally distributed data, respectively. The local outlier factor (LOF) method was employed for outlier detection. We found no outliers in any of our data. *P* values of less than 0.05 were considered statistically significant. All of the statistical details of experiments can be found in the figure legends, including the statistical tests used and the exact value of n, which represents the number of individual animals. All data are expressed in mean ± s.e.m. The sample size for animal studies was determined based on our previous experience with similar experiments and not on a statistical method. These animal studies was not performed in a blinded manner, and the investigators were aware of the group allocation during the experiment and when assessing the outcome.

## Results

### *Apoe* expression is significantly decreased in PBMCs of PAE mice in association with their impaired motor learning

**Fig. 1:**
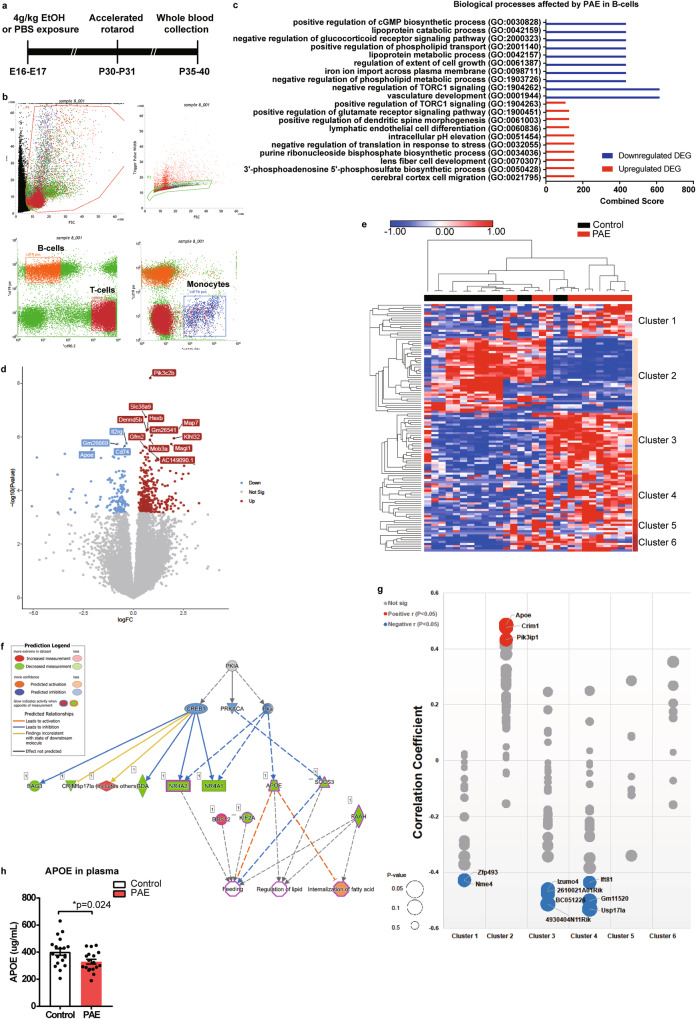
PAE reduces APOE expression in PBMCs. **a** Schematic of PBMC RNA profiling following accelerated rotarod test. **b** A representative result of FACSorting of PBMCs collected from control animals at P35. PBMCs were labeled with CD11b, CD19, and CD90.2, and sequentially sorted based on the cell size (forward scatter [FSC] versus side scatter [SSC]) and singlets (FSC vs. trigger pulse width). Then cells were further divided into B-cells (CD19+/CD90.2−), T-cells (CD90.2+/CD19−), and monocytes (CD11+/CD19−) indicated by orange, red, and blue boxes, respectively. **c** Top 10 enriched gene ontologies (GOs) with Combined Scores (see “Materials and methods”) in downregulated (blue) and upregulated (red) differentially expressed genes (DEGs) by PAE in B-cells. **d** Volcano plot shows the DEGs by PAE in B-cells. Top 15 DEGs are labeled with their gene symbols. Blue and red indicate downregulated and upregulated genes, respectively. **e** Heatmap of hierarchical K-means clustering shows 6 distinctive clusters of DEGs by PAE in B-cells. Gene names in each cluster are shown in Supplementary Table 2. DEGs in cluster 2 contains lipid metabolism-related GOs (Supplementary Fig. 1c). **f** Result of IPA on the genes in cluster 2. Regulator effects network overlaid with top diseases and functions shows that decreased *Apoe* expression is associated with other genes that have reduced expression by PAE and fall in the same pathways. **g** Bubble plot of Pearson’s correlation analysis between the gene expression level and learning index of animals (control: n = 15, PAE: n = 14). Red and blue bubbles indicate significant (*P* < 0.05) positive and negative correlations, respectively. Grey bubbles indicate non-significant (*P* ≧ 0.05) correlations. The size of the circle corresponds to the p-value. **h** PAE mice show significantly lower levels of plasma APOE than control mice (control: n = 19; PAE: n = 18). *P = 0.024 by two-tailed Student’s *t*-test. Data represent mean ± s.e.m.

Growing evidence suggests that gene transcription measured in human blood correlates with transcripts measured in many other body systems, including the brain [39, 40], indicating potential utilization of peripheral genomic information to decipher molecular changes in the brain which is difficult to get samples from. In fact, a previous study has demonstrated that genomic loci that are differentially methylated between PAE and control mice in the hypothalamus are highly similar to those in PBMCs [41]. Therefore, we performed RNA sequencing of PBMCs from PAE and control mice to gain insights into the molecular changes in the brain attributable to PAE. Ethanol or PBS vehicle was administered to pregnant dams at embryonic day (E) 16 and 17 to generate PAE mice, as an animal model of FASD, and control mice, respectively (Fig. 1a, details in Materials and methods). This PAE regimen does not induce gross brain structural changes but elicits fine and gross motor learning deficits in mice [13]. Following the generation of PAE and control mice, an accelerated rotarod test was performed on postnatal day (P) 30 as previously done [13] and whole blood was collected the on P31 to isolate PBMCs. Using fluorescence-activated cell sorting (FACSorting) with antibodies for CD19, CD90.2, and CD11b, B- and T-cells and monocytes were sorted, respectively (Fig. 1b).

With limma-voom algorithms [19], differentially expressed genes (DEGs) between control and PAE mice were defined with a cut-off of twofold change and *p* value < 0.05. We found no DEGs in monocytes, while 116 (35 downregulated and 81 upregulated in PAE) and 6,568 (178 upregulated and 6,390 downregulated in PAE) DEGs were found in B-cells and T-cells, respectively. Of note, enrichment analysis (enricher) showed that downregulated DEGs in B-cells were enriched in lipoprotein metabolism processes and phospholipid-related pathways (Fig. 1c and Supplementary Table 1). Among genes in these pathways, *Apoe*, which serves the major function of lipoprotein metabolism [42], was one of the top genes that were significantly downregulated in PAE mice (Fig. 1d). We also performed K-means clustering of DEGs in B-cells by using the optimal number of clusters determined by the Within Sum of Squares method (Supplementary Fig. 1a). As a result, DEGs were classified into 6 distinctive gene clusters (Fig. 1e and Supplementary Table 2). Gene Ontology (GO) enriched in each cluster was determined (Supplementary Fig. 1b–g). In cluster 2 that included *Apoe*, lipid-related pathways such as cholesterol transporter activity and lipoprotein particle receptor binding were enriched (Supplementary Fig. 1c). Similarly, Ingenuity Pathways Analysis (IPA) of cluster 2 suggested changes in the regulation of lipid and internalization of fatty acids in PAE mice (Fig. 1f).

Our previous study found that PAE caused motor learning deficits in mice [13]. Therefore, a correlation analysis between the expression of the DEGs and the motor learning index (see “Materials and methods“) in PAE mice was conducted. We found that *Apoe* expression in cluster 2 demonstrated the highest (positive) correlation with the motor learning index (Fig. 1g). *Crim1*, which encodes a transmembrane protein containing an insulin-like growth factor-binding domain and plays a role in organ development [43], and *Pik3ip1*, which encodes a protein that negatively regulates phosphatidylinositol 3-kinase (PI3K) to control neuronal survival and metabolism [44], were also significantly correlated with motor learning. Both were also from cluster 2 (Fig. 1g).

In T-cells, enrichment analysis also revealed that downregulated DEGs were enriched in lipid response, fat differentiation, MAP kinase, and cytokine signaling pathways (Supplementary Fig. 2a). No significant GOs were identified with the list of upregulated DEGs in T-cells. The top regulator effect network identified by IPA included changes in lipid synthesis (Supplementary Fig. 2b). As both B- and T-cells showed changes in lipid-related pathways in their downregulated gene sets by PAE, we examined the genes commonly downregulated between B- and T-cells and found that *Apoe* was one of six such genes (Supplementary Fig. 2c). Consistent with the result of RNA sequencing, we also found that the level of circulating APOE in the plasma was significantly decreased in PAE mice compared to control mice at P30 (Fig. 1h).

### APOE expression in the motor cortex is reduced in PAE mice

**Fig. 2:**
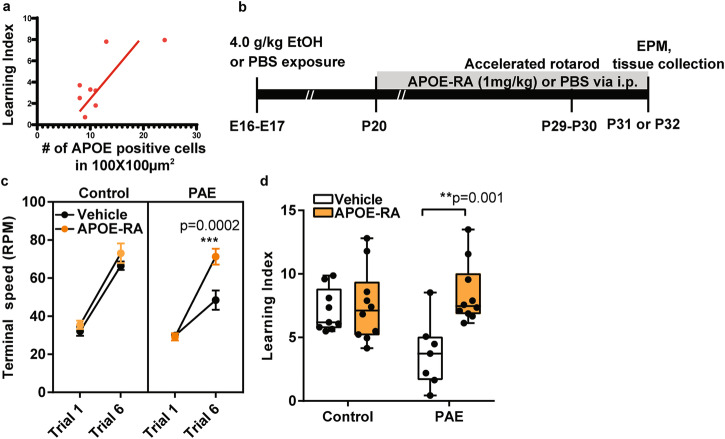
APOE-RA improves motor learning in PAE mice. **a** Pearson’s correlation analysis reveals a positive correlation (*R*^*2*^ = 0.558, *P* = 0.0166) between the motor learning index and the number of APOE positive cells in the motor cortex in PAE mice (Supplementary Fig. 3). Each dot represents an individual animal (n = 8). Solid line indicates the best fit line. **b** Schematic shows a timeline of the APOE-RA treatment and behavioral tests. **c** PAE mice treated daily with APOE-RA for 10 days show significantly improved terminal speed in trial 6 in accelerated rotarod test compared to vehicle-treated PAE mice (control+vehicle: n = 9; control+APOE-RA: n = 10; PAE+vehicle: n = 7; PAE + APOE-RA: n = 10). ***P < 0.001 by two-way ANOVA with simple main effect test. Data represent mean ± s.e.m. **d** Postnatal APOE-RA treatment significantly improves motor learning index in PAE mice (control+ vehicle: n = 9; control+APOE-RA: n = 10; PAE+vehicle: n = 7; PAE + APOE-RA: n = 10). Box plot represents 25th, median, and 75th percentile. Whiskers extended to min and max values. **P < 0.01 by two-way ANOVA with simple main effect test. Data represent mean ± s.e.m.

Based on the finding of an association between decreased APOE in peripheral blood and impaired motor learning in PAE mice, we next examined APOE expression in the motor cortex, one of the brain regions primarily responsible for motor learning. We observed puncta-like expression of APOE by immunohistochemistry in normal mice consistent with previous reports [45–48], and confirmed no immunolabeling by the negative control procedure without including the primary antibody. Immunohistochemistry at P30 revealed a significant reduction of APOE-positive cells in the motor cortex in PAE mice compared to control mice (Supplementary Fig. 3a, b). The adjacent cingulate cortex showed no difference in the number of APOE-positive cells between control and PAE mice (Supplementary Fig. 3a). Correlation analysis further revealed that the number of APOE-positive cells in the motor cortex was positively correlated with the motor learning index in PAE mice, indicating that animals with fewer APOE-positive cells have poorer motor learning (*R*^2^ = 0.56, *P* = 0.017) (Fig. 2a), consistent with the result of correlation analysis on B-cells (Fig. 1g).

Previous studies have shown that APOE is expressed in both neurons [49, 50] and astrocytes [51] in the brain. Double immunolabeling for APOE with neuronal nuclei (NeuN) or glial fibrillary acidic protein (GFAP) in both control and PAE mouse brain sections revealed that APOE was expressed in both NeuN-positive neurons and GFAP-positive astrocytes in the entire layer V/VI of the motor cortex (Supplementary Fig. 3c, e). We previously observed no obvious changes in the brain structure and neuronal number in PAE mice [13]. Similarly, we found no significant difference (*P* = 0.34) in the number of GFAP-positive astrocytes between control and PAE mice (Supplementary Fig. 3g, h), suggesting that the reduction in the number of APOE-positive cells in the brain of PAE mice is not due to changes in the number of astrocytes or neurons, but due to reduction in the APOE expression level in these cells.

Since a recent study using a similar PAE mouse model has demonstrated that PAE induces a small but significant increase in a specific type of apoptosis in the developing brain [52], we further investigated whether the reduction in the number of APOE-positive cells in PAE mice is due to the loss of cells with high levels of APOE expression (Selective Cell Loss model) or APOE expression levels are decreased overall in all cells (Uniform Reduction model). We first confirmed that both NeuN+ neurons and GFAP+ astrocytes show reduction of APOE immunolabeling in the motor cortex in PAE mice (Supplementary Fig. 3c–f). To determine which model aligns with the case of PAE mice, we created two simulated datasets (Supplementary Fig. 3d, f). The simulated dataset corresponding to the Selective Cell Loss model was formulated by excluding data points of top 16 neurons and top 11 astrocytes with the highest APOE levels from the data of control (PBS-exposed) mice. These thresholds were set so that the mean APOE level values of these simulated datasets matched those of the data obtained from PAE mice. The datasets corresponding to the Uniform Reduction model were created by multiplying the APOE level data of control mice by a fixed ratio so that their mean APOE level values matched those of the data of PAE mice. Comparison of Cohen’s *d* values relative to the data of control mice between the actual data of PAE mice and the simulated data of two models suggested that the APOE reduction in PAE mice is likely due to a uniform decrease in APOE expression in many cells rather than to the loss of high APOE-expressing cells, for both neurons and astrocytes (Supplementary Fig. 3d, f). However, these results do not completely exclude the possibility that the uniform reduction of APOE may encompass an adaptive response to PAE-induced cell death, given the role of APOE in neuronal death [53].

### APOE-RA improves motor learning and reduces anxiety in PAE mice

The PBMC RNA sequencing and immunohistochemical analysis of the PAE model supported the possibility that the hypoexpression of APOE by PAE in the brain could be involved in neurobehavioral changes in PAE offspring. In fact, *Apoe* knockout mice exhibit spatial [10] and reversal learning deficits [51] similar to those shown in another PAE mouse model and PAE patients [54, 55]. One of the major APOE receptors, LRP1, is located at post-synaptic sites, and abolishing these receptors in neurons results in impaired motor function in mice [12], suggesting that the APOE-LRP1 interaction is required for proper motor function.

To examine whether application of APOE mitigates neurobehavioral deficits in PAE mice, we tested APOE-RA (or COG 133), which contains the receptor binding unit of human *APOE* and binds to *LRP1*, but does not include the lipid-binding domain [56]. While full-length APOE does not cross the blood-brain barrier (BBB) [57], APOE-RA crosses it [58]. Therefore, we first checked whether administered APOE-RA reached the target neurons in the motor cortex where we saw significant reduction of APOE-positive cells in PAE mice (Supplementary Fig. 3a, b). Biotinylated APOE-RA was injected intraperitoneally (i.p.) at 1.0 mg/kg body weight at P20, and brains were collected 5 minutes after the administration. Modified streptavidin-biotin staining detected the binding of APOE-RA to cells in the entire motor cortex, including LRP1-expressing cells in both layer II/III and layer V/VI (Supplementary Fig. 4). No significant correlation was found between the levels of biotinylated APOE-RA detection and (endogenous) APOE staining in individual cells in layer V/VI in PAE mice, suggesting that the effects of APOE-RA binding was not different among cells with different levels of endogeneous APOE expression (Supplementary Fig. 4f, g). The biotinylated APOE-RA was also detected in other brain areas, including the cingulate cortex (Supplementary Fig. 5a, b). We confirmed that BBB integrity was not compromised in PAE mice using two different dyes, sodium fluorescein, and Evans blue, at P30 (Supplementary Fig. 6a–d).

To test the effect of APOE-RA on learning deficits in PAE mice, it was administered i.p. daily at 1.0 mg/kg body weight from P20 to P31 or 32 as depicted in Fig. 2b. First, the accelerated rotarod test was performed around P30. We found that motor learning deficits in PAE animals were significantly improved by APOE-RA administration; the terminal speed at the last trial (trial 6) and the learning index were significantly higher in APOE-RA-treated PAE mice compared to vehicle-treated PAE mice (Fig. 2c, d). On the other hand, no significant differences were detected in motor learning behavior in control mice between APOE-RA and vehicle treatment, or in the body weight during the treatment between control and PAE mice (Supplementary Fig. 6e, f).

24 h after the accelerated rotarod test, the same animals were placed on an elevated plus maze to assess anxiety. A previous study demonstrated that PAE mice spent a smaller percentage of total time in open arms than control mice [14]. Consistently, the percentage of time spent in open arms by PAE mice was shorter than that by control mice without treatment, but the percentage was brought back to the control level by APOE-RA treatment (Supplementary Fig. 5c, d), suggesting that the anxiety behavior in PAE mice was also mitigated by APOE-RA treatment. In control mice, there was no APOE-RA effect on the open arm time (Supplementary Fig. 5c, d). Overall, these data demonstrate that decrease of APOE contributes to the motor learning deficit and anxiety behavior in PAE mice, and administration of APOE-RA positively mitigates both behavioral abnormalities.

### Decrease of APOE by PAE alters NMDAR function and upregulates KCNN2 expression

Next, we examined the molecular pathways affected by reduction of APOE underlying neurobehavioral abnormalities in PAE mice. In the motor cortex at P30, we examined the effects of PAE on the expression levels and patterns of LRP1, as well as the NMDA receptor (NMDAR) and PSD-95, for which interactions with LRP1 at the postsynapse have been demonstrated [59, 60]. GluN1 antibody was used to label NMDARs as GluN1 is an obligatory subunit for the receptor [61] and its expression in the motor cortex was shown to be positively associated with motor learning in mice [62]. However, we found no significant changes in any of those proteins (Supplementary Fig. 7a–f), suggesting that PAE affects the neurobehavioral deficits by mechanisms other than the expression of these APOE-related synaptic proteins.

Our previous study found that the expression of potassium intermediate/small calcium-activated channel, subfamily N, member 2 (KCNN2) was upregulated in the motor cortex of PAE mice, and knockdown of *Kcnn2* expression in the motor cortex or pharmacological inhibition of KCNN2 function improved the motor learning deficits in PAE mice [13]. In the mouse model of Angelman syndrome that shows motor learning deficit and anxiety similar to our mouse model of FASD [63–65], malfunction of the feedback regulation between NMDA and KCNN2 pathways was shown to lead to excessive KCNN2 at synapses and cause intellectual disabilities [66]. Other studies have also shown a functional regulation of NMDARs by LRP1 receptor activation through their coupling via PSD-95 [59, 67]. Therefore, we hypothesized that changes in LRP1-mediated NMDAR function due to reduced APOE underlie the increased expression and/or synaptic distribution of KCNN2 in PAE mice.

**Fig. 3:**
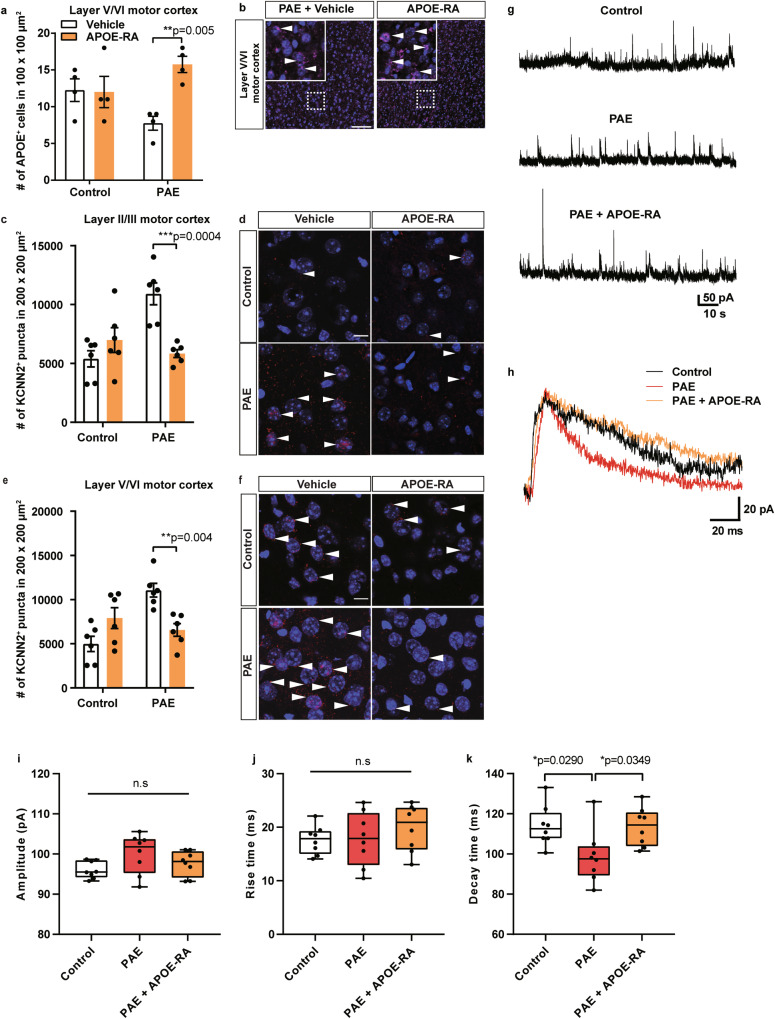
Postnatal APOE-RA treatment alleviated the decreased endogenous APOE level, excessive KCNN2, and shorter decay time of NMDAR-mediated sEPSC in the motor cortex in PAE mice. **a** APOE-RA treatment increases the number of APOE-positive cells that is reduced by PAE in layer V/VI of the motor cortex (n = 4 per group). **P < 0.01 by two-way ANOVA with simple main effect test. Data represent mean ± s.e.m. **b** Representative images of APOE (magenta, arrowheads) and DAPI (blue) staining in layer V/VI of the motor cortex in PAE mice treated with vehicle or APOE-RA. Dotted boxes indicate the region shown at higher magnification in the inset. The number of KCNN2 puncta is significantly decreased in layer II/III (**c**) and layer V/VI of the motor cortex (**e**) in PAE mice after the APOE-RA treatment compared to vehicle treatment (n = 6 per group). **P < 0.01, ***P < 0.001 by two-way ANOVA with simple main effect test. Data represent mean ± s.e.m. **d**, **f** Representative images of KCNN2 (red, arrowheads) and DAPI (blue) staining. Scale bars = 10 µm. **g** Examples of NMDAR-mediated sEPSCs recorded under the voltage-clamp condition at a holding potential of +50 mV in pyramidal neurons of layer V/VI in the indicated experimental groups. **h** NMDAR-mediated sEPSCs averaged from 5 events in control, PAE, and PAE + APOE-RA mice. **i–k** No significant differences are observed in the amplitude (**i**) and rise time (**j**) between the groups. PAE group shows a significantly shorter decay time than control and APOE-RA treated PAE group (**k**). Box plot represents the 25th, median, and 75th percentile. n = 8 cells per group. Whiskers extend to min and max values. *P < 0.05 by One-way ANOVA with Tukey’s post hoc test.

To test this hypothesis, we first examined whether excitatory synaptic transmission via NMDARs and their kinetics are changed in PAE mice. Spontaneous NMDA excitatory postsynaptic currents (sNMDA EPSCs) were recorded in pyramidal neurons in layer V/VI of the motor cortex at P30. There was no difference in the amplitude and rise time in sNMDA EPSCs in PAE mice compared to control mice (Fig. 3g-j), indicating no changes in the synaptic response through NMDARs. However, we found a significantly shorter decay time in sNMDA EPSCs in PAE mice compared to control mice (Fig. 3h, k). Consistent with the altered NMDAR kinetics, we observed reduced downstream phosphorylation of cAMP-response element binding protein (CREB) [68, 69] in the motor cortex of PAE mice (Supplementary Fig. 8).

Treatment with APOE-RA for 10 days starting from P20 restored the shorter decay time in PAE mice to the level comparable to control mice (Fig. 3k), with no significant effects on the amplitude and rise time (Fig. 3i, j). Increased expression of KCNN2 in the motor cortex by PAE [13] was also restored to the control levels after APOE-RA treatment (Fig. 3c–f). Double immunohistochemistry with KCNN2 and PSD-95 revealed the reduction of the PAE-induced increase in postsynaptic KCNN2 levels after the APOE-RA treatment (Supplementary Fig. 7g–i). These results suggest that reduced APOE contributes to motor learning deficits in PAE mice by increasing KCNN2 expression and its synaptic level via altered NMDAR function.

The decay time of sNMDA EPSCs is thought to shift from slow to fast during development by the change in the ratio between two NMDAR subunits GluN2A and GluN2B [70]. However, no significant difference was observed in the expression of *Grin2a* and *Grin2b* (GluN2A and GluN2B encoding genes, respectively) in the motor cortex between control and PAE mice (Supplementary Fig. 9e–j).

### Decrease in brain APOE in PAE mice involves regulation at the transcriptional level, while APOE-RA increases brain APOE at the post-transcriptional level

As we observed reduction of *Apoe* mRNA in PBMC (Fig. 1) and APOE protein in the motor cortex of PAE mice (Fig. 3a, b), we next examined the expression of *Apoe* mRNA in the motor cortex of PAE mice. By utilizing a specific RNAscope probe, *Apoe* mRNA was detected around the nucleus (Supplementary Fig. 9c), while a negative probe provided no signals as expected (Supplementary Fig. 9d). Consistent with the reduced protein levels, reduction in the *Apoe* mRNA expression was observed in the motor cortex in PAE mice (Supplementary Fig. 9a-c).

A previous study has shown that Ac-hE18A-NH_2,_ an APOE mimetic peptide that has a similar structure to APOE-RA, increases endogenous brain APOE levels [71]. Thus, we also tested whether endogenous APOE expression, which is decreased in the brain in PAE mice (Fig. 3 and Supplementary Fig. 3), was changed by APOE-RA administration. Based on the half-lives of similar APOE mimetic peptides [Ac-hE18A-NH_2_: <2 minutes for fast decay time and 90 min for long decay time [72]; COG1410: 13 ± 5 min [73], we assumed that most of the APOE detected 24 h after the last dose of APOE-RA to be de novo endogenous APOE expression. Immunolabeling with an APOE antibody 24 hours after the final administration of the 10-day APOE-RA treatment showed an increase in APOE-positive cells in layer V/VI of the motor cortex in PAE mice compared to vehicle-treated PAE mice at P30 (Fig. 3a, b). A similar increase in APOE-expressing cells by APOE-RA treatment was also observed in the cingulate cortex in PAE mice (Supplementary Fig. 5e, f). In control mice prenatally exposed to PBS, we confirmed no significant change in endogenous APOE expression by APOE-RA treatment in the motor and cingulate cortices (Fig. 3a and Supplementary Fig. 5e). In contrast to the recovery of APOE protein level in PAE mice after APOE-RA treatment, no significant effect on the *Apoe* mRNA level was observed (Supplementary Fig. 9a–c). Together, these results indicate that the reduction of brain APOE level in PAE mice involves its reduction at the transcriptional level, while APOE-RA treatment may increase the endogenous APOE level in PAE mice at the posttranscriptional level.

### Chromatin accessibility at the *ApoE* locus is decreased in motor cortex of PAE mice

**Fig. 4:**
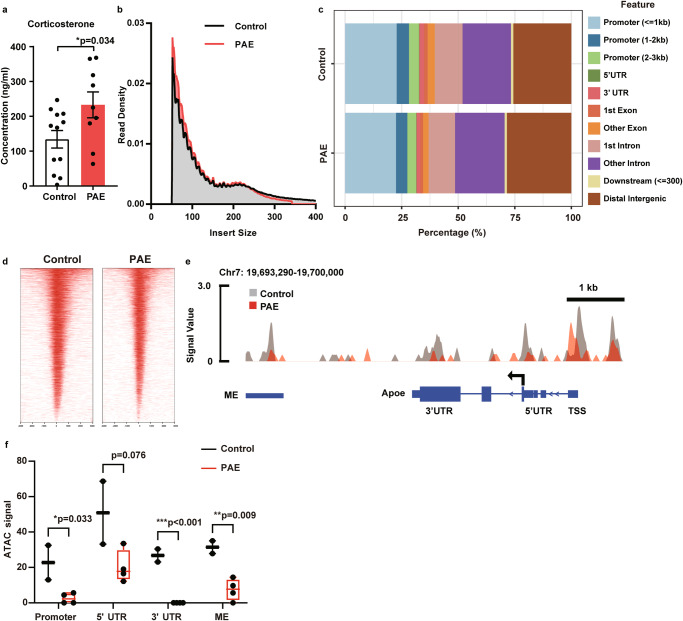
PAE decreases chromatin accessibility in the brain. **a** PAE mice show a significantly increased corticosterone concentration in the plasma compared to control mice (control: n = 12, PAE: n = 9). *P < 0.05 by two-tailed Student’s t-test. Data represent mean ± s.e.m. **b** Distribution of ATAC-seq fragment lengths shows enrichment under 100 bp and around 200 bp, indicating nucleosome-free and mono-nucleosome-bound fragments, respectively. **c** Peak annotation graphs show that the proportion of aligned genomic features is similar between control and PAE samples. **d** Heatmap shows the enrichment of ATAC reads around the transcription start site (TSS) (−1,000- + 1,000) in both control and PAE mouse samples. ATAC-seq signal in regulatory regions of the *Apoe* (**e**) and the quantification (**f**) show significantly reduced chromatin accessibility in the promoter, 3’ untranslated region (UTR), and multienhancer (ME) in the PAE group. 5’ UTR shows a trend of decrease in the PAE group (control: n = 2, PAE: n = 4). *P < 0.05, **P < 0.01,***P < 0.001 by two-tailed Student’s *t*-test. The box plot represents the 25th, median, and 75th percentile. Whiskers extend to the min and max values. Each dot represents an individual animal.

The *Apoe* regulatory region contains multiple glucocorticoid receptor binding loci [74, 75], and an increase in glucocorticoids enhances *APOE* gene expression by the direct binding on these loci [74]. In FASD children, the hypothalamic-pituitary-adrenal (HPA) axis has been shown to be hyperactive and increase cortisol levels [76]. Similarly, our PAE mice showed increased plasma corticosterone levels (Fig. 4a), which contradicted the decreased *Apoe* mRNA level in the brain (Supplementary Fig. 3).

A previous study demonstrated that chromatin structure changes in response to PAE and that these changes persist beyond the window of exposure [77]. Therefore, we hypothesized that *Apoe* gene transcription is epigenetically silenced by PAE. To test chromatin opening, we performed the assay for transposase-accessible chromatin with sequencing (ATAC-seq) of pyramidal neurons in the motor cortex of PAE and control mice. Neurons labelled with GFP introduced by *in utero* electroporation at E15 were collected by FACSorting. After Tn5 enzymatic raction, we confirmed the majority of fragment sizes corresponded to nucleosome-free regions (<100 bp) and mono-nucleosome (~200 bp) (Fig. 4b). Peak annotations were comparable between control and PAE groups (Fig. 4c). The density map also showed that ATAC peaks were enriched near the transcription starting site (TSS), confirming the quality of ATAC-seq (Fig. 4d). We then quantified and compared the signals around the *Apoe* locus between PAE and control. A significant reduction of ATAC signals was found in the promoter, 3′ UTR, and the multienhancer (ME), which controls *Apoe* promoter activity in different cell types [78], in the PAE group (Fig. 4e, f), suggesting that the chromatin accessibility was reduced, and thereby *Apoe* expression was reduced in the motor cortex of PAE mice (Supplementary Fig. 9a). These results indicate that the reduction of *Apoe* expression in the motor cortex is attributed to epigenetic effects of PAE.

### A SNP in the APOE enhancer is associated with impaired neurocognition in human subjects in the presence of PAE

**Fig. 5:**
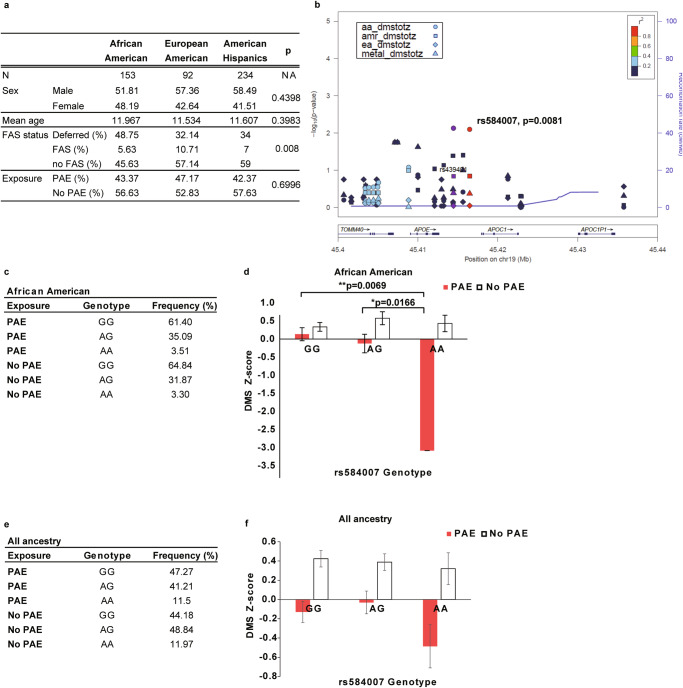
The SNP rs584007 in an *ApoE* enhancer is associated with DMS Z-score in PAE children. **a** Table shows demographic information on study subjects. No significant differences are found in the gender ratio and mean age between the three ancestry groups by Chi-square test and one-way ANOVA, respectively. A significant difference in fetal alcohol syndrome (FAS) diagnosis, but not in the number of PAE individuals, is observed between the ancestry groups by Chi-square test. NA = not applicable. **b** Regional plot between 45.4 and 45.44 Mb on chromosome 19 around the *APOE* locus in the African (aa), American Hispanic (amr), and European (ea) ancestries and meta-analysis (metal) of GWAS with DMS Z-score. The interaction between SNP and PAE shows a significant association with the DMS z-score in the group of African American ancestry. **c** Table indicates the frequency of individuals with the rs584007 genotype in the African American ancestry group. **d** In the group of African American ancestry, AA individuals prenatally exposed to alcohol have significantly lower DMS Z-scores compared to individuals with other genotypes with or without exposure to alcohol. *P < 0.05, **P < 0.01 by two-way ANOVA with simple main effect test. Data represent mean ± s.e.m. **e** Table indicates the frequency of individuals with the rs584007 genotype in all ancestry groups. **f** When all ancestry groups are combined, no significant interaction between PAE and SNP is observed by two-way ANOVA (*P* = 0.3769). A significant alcohol effect is observed (*P* < 0.0001). There is a marginal difference in the DMS Z-score between AA and other genotypes in alcohol exposed groups (AA vs GG *P* = 0.09937; AA vs AG *P* = 0.0991 by Tukey’s post hoc test). Data represent mean ± s.e.m.

In humans, APOE level in the cerebrospinal fluid is known to be affected by genetic variants [79]. Therefore, we tested whether common variants (MAF ≥ 0.01) around the *APOE* locus between 45.40 and 45.55 Mb on chromosome 19 were associated with neurobehavioral traits of PAE individuals genotyped as part of a genome-wide association study (GWAS). Saliva samples were collected from children with prenatal alcohol exposure and controls with minimal or no prenatal alcohol exposure of three ancestry groups (African Americans, European Americans, and American Hispanics) in the Collaborative Initiative on Fetal Alcohol Spectrum Disorders (CIFASD) US cohort (for more information on CIFASD methods, see [80, 81]). There were no significant differences between these three ancestry groups in the sex ratio, mean age, and presence of PAE (Fig. 5a). Within the group with PAE, which was recruited for participation in a study on the effects of PAE, the prevalence of FAS ranged 5–10%, with European Americans being the highest (Fig. 5a). While this prevalence is much higher than one previously reported [82], CIFASD was not designed as a prevalence study and individuals with PAE were specifically recruited for participation.

The GWAS analysis revealed a significant (*P* = 0.008) association between rs584007 genotype and PAE with the delayed matching to sample (DMS) Z-score in African Americans with PAE (aa_dmstotz in Fig. 5b). This SNP is located in the multienhancer (ME), where we found that chromatin was closed by PAE in the mouse motor cortex (Fig. 4f). The DMS assessment is a cognitive test that measures short-term visual recognition memory, and a human fMRI study demonstrated that the task requires activation of both the prefrontal cortex, including the cingulate cortex, and the premotor area, which play important roles in decision-making and motor control, respectively [83]. Within the group of African Americans with PAE, children with at least one A allele scored lower than GG. Although number of subjects is low, we found the genotype with a homozygous for rs584007 SNP (AA) interacts with PAE to yield the lowest performance in DMS test (Fig. 5c, d). When analysis was done between the rs584007 SNP genotype and DMS Z-score data of all three ancestry groups, a marginal reduction in the Z-score of AA-carrying PAE individuals was found (Fig. 5e, f). Allele frequency is not significantly different between ancestry groups (Supplementary Table 3). Together, these data suggest that PAE children who carry the SNP within the ME region have higher risk of low DMS Z-scores.

### Reduced plasma APOE level is associated with lower cognitive performance in infants with PAE

**Fig. 6:**
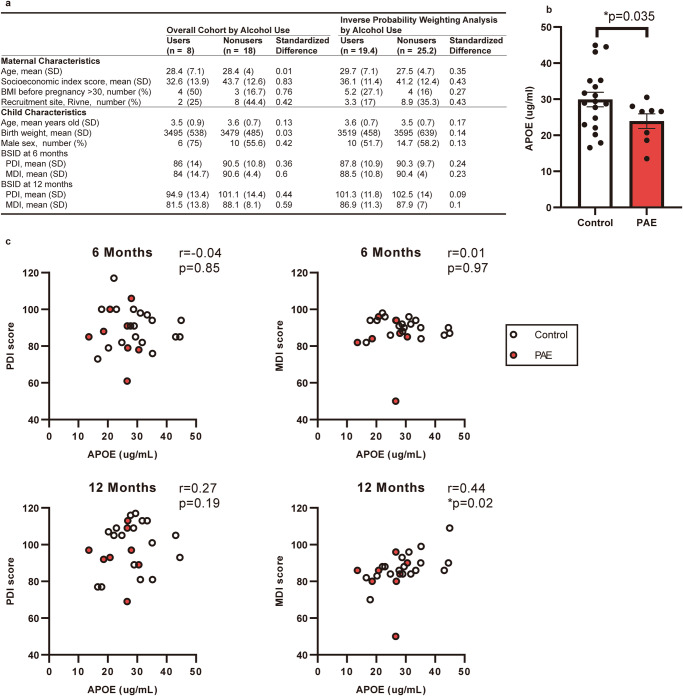
APOE in the plasma is reduced in PAE patients. **a** Baseline characteristics of mothers who drank alcohol during pregnancy (users) and who did not (nonusers) and their children. Right columns show the results of Inverse probability weighing analysis. **b** Graph shows that the plasma APOE level at age 2–4 years old is significantly lower in PAE children. *P < 0.05 by Student’s t-test. Data represent mean ± s.e.m. **c** Pearson’s correlation analysis shows positive correlations between the plasma APOE level and the BSID-II MDI score at 12 months old, but not at 6 months old.

In an independent CIFASD cohort in Ukraine, plasma samples were collected from children with PAE and without aged 2–5 years old. In these subjects, we examined plasma APOE levels and the relationship between these levels and neurodevelopmental outcomes. By inverse probability weighting (IPW) with 4 maternal variables (age, socioeconomic index score, BMI before pregnancy, and recruitment site) and 4 child variables (age, birth weight, sex, and BSID-II) between two groups (Fig. 6a), we found that plasma APOE levels in children with PAE were significantly lower than those in controls (Fig. 6b), as was observed in mice with PAE. Children in this cohort were also previously assessed for neurodevelopmental delay between 6 and 12 months of age, using the Bayley Scales of Infant Development (BSID-II), and specifically, the Psychomotor Development Index (PDI) and Mental Development Index (MDI). Pearson’s correlation analysis indicated a significant correlation between child plasma APOE levels and previously obtained infant MDI scores (Fig. 6c).

## Discussion

Utilizing a mouse model of FASD, our study discovered that APOE is a potential target for treatment and a peripheral biomarker for neurobehavior problems in FASD. Transcriptomics and immunohistological assays revealed a reduction of both peripheral and brain APOE in mice with PAE (Figs. 1, 2 and Supplementary Fig. 3). ATAC-seq data suggest that the reduction of chromatin accessibility around the *Apoe* locus in PAE mice is responsible for the reduction of APOE in the brain (Fig. 4). The positive correlation between the APOE level and motor learning suggests that the decrease in APOE level contributes significantly to the deficits in motor skill learning in PAE mice (Fig. 1g, 2a). Similarly, we found that the plasma APOE level was reduced in PAE children, and the level was positively correlated with BSID-II scores at 12 months old (Fig. 6). A peptide, APOE-RA, which mimics the APOE function as a ligand for its receptors, alleviated both the motor learning deficit and anxiety in PAE mice (Fig. 2 and Supplementary Fig. 5), demonstrating that appropriate levels of APOE receptor-mediated signaling are essential for normal performance in various behaviors.

While a blood alcohol concentration (BAC) was not measured in our Ukrainian cohort, a previous study has suggested that the BAC in pregnant individuals engaging in binge drinking, which is defined as consuming more than five standard drinks per occasion (same as our Ukrainian cohort), ranges from 110 to 370 mg/dl [84]. Pregnant mice in the current study showed the BAC of 198–410 mg/dl within an hour after alcohol administration [85], therefore, are expected to serve as a good model for these human subjects. However, since i.p. injection was used for alcohol administration to achieve precise dose control, these animals may have achieved target BAC levels more rapidly than human subjects [86]. Species differences, including that in the placental structure [87], may also cause differences in alcohol metabolism during pregnancy.

We observed significant correlation of plasma APOE level with MDI but marginal correlation with PDI (Fig. 6c). We also detected marginal reduction of MDI score in PAE group at 6- (*P* = 0.09 by Student’s t-test) and 12-month-old (*P* = 0.14), but not in PDI (*P* = 0.38, 0.31 at 6, 12 month-old, respectively). As seen in our mouse PAE model [13] (Fig. 2c at trial 1), this data suggests that gross motor skill may be largely unaffected by PAE. As MDI shows higher correlation with cognitive indices at later stages in general population [88], the deficits in motor skill learning seen in mouse juvenile with PAE may be a consistent result with lower MDI in human infants with PAE. Lower APOE level was also observed in plasma of both mouse model and human children with PAE. The moderate correlation between the APOE level and MDI may reflect the limited number of subjects and/or the temporal difference in data collection (BSID-II was conducted in infancy, while plasma APOE levels were measured between 2 and 5 years old). Follow-up studies on later neurodevelopmental testing with these children is ongoing to further clarify the relationship between APOE and neurodevelopment.

The DMS assessment, a cognitive test that measures short-term recognition memory, is affected by interaction of a variant in APOE enhancer and PAE (Fig. 5d). Ablation of hippocampus does not change the performance in animals [89], while other studies show contribution of various cortical and subcortical regions including premotor, primary motor and prefrontal cortices [83, 90]. These results suggest the contribution of broader circuitry in DMS task, and that the molecular mechanisms observed in motor cortex of mouse PAE may be applicable to other brain regions, altogether leading to the cognitive problems.

Paucity in data in human FASD cohort also limits our interpretation, especially APOE’s contribution in anxiety phenotypes in mice with PAE. Consistent with our observation (Supplementary Fig. 5c, d), both increase of cortisol and anxiety had been observed in PAE human subjects as well as various animal models of PAE [76, 91, 92]. Although previous study showed crucial contribution of reduced neuronal activities in the anterior cingulate cortex in anxiety phenotype in the mouse with PAE [14], the mechanism how APOE-RA affect activities in ACC to improve the phenotype remain still elusive.

Our knowledge of the genetic factors that underlie vulnerabilities to FASD and alcohol teratogenicity is still in its infancy. Animal research and human epigenetic studies have shown that maternal and fetal genetic factors contribute to teratogenic risk of alcohol [93–95]. Although the number of subjects is small as a nature of FASD cohort, our GWAS study suggests that the rs584007 SNP interacts with PAE in reducing DMS Z-scores in children (Fig. 5d, f). The rs584007 SNP is located in the ME of *ApoE* locus where we found the chromatin is closed by PAE in mice (Fig. 5f).

ME region is known to crucially control the transcription of *APOE* in humans [75, 96, 97]. Variants in the ME, including rs584007, have been shown to affect the *APOE* promoter activity and moderately decrease the APOE level in the cerebrospinal fluid in human adults [79, 98]. Furthermore, ME has been shown to physically interact with the *APOE* promoter in an anti-sense orientation in human macrophages and monocytes [78], suggesting that variants in the ME may affect *APOE* promoter activity through direct interactions with the promoter to modulate *APOE* expression. These previous studies and the present results collectively suggest that the genetic predisposition in the *APOE* enhancer exacerbates PAE-caused reduction of APOE expression, and thereby increases the risk of eliciting neurobehavioral problems.

Besides transcriptional regulation at the *APOE* locus, other mechanisms are possibly involved in APOE expression and distribution. For instance, a paper has reported that the choroid plexus/CSF provides an additional source of APOE, and the glymphatic fluid transporting system delivers APOE to the brain via the periarterial space [99]. Interestingly, reduced inflow of APOE-containing CSF by sleep deprivation has been shown [99]. As sleep deprivation in FASD patients has been reported [100, 101], this mechanism may be involved in APOE reduction by PAE.

The APOE level was decreased in the motor cortex but not in the cingulate cortex in PAE mice (Fig. 2a and Supplementary Fig. 3). Region specific regulation of endogenous APOE, e.g., high expression of *APOE* mRNA in neurons in the frontal cortex and hippocampus while little expression in the cerebellar cortex in the human brain [49], may underlie such region specific alteration by PAE. As we used an acute prenatal exposure model, it is possible that the regional specificity also depends upon the timing and dosage of alcohol exposure. The correlation between the APOE level in the motor cortex and motor learning index (Fig. 2a) is consistent with the fact that the motor cortex plays a crucial role in motor learning [102–105]. Mice with PAE exhibit anxiety behavior, which is attributed to the reduced activity of the cingulate cortex [14]. Although increased expression of endogenous APOE was observed in the cingulate cortex in mice with PAE by APOE-RA administration, which mitigated the anxiety behavior (Supplementary Fig. 5), no significant decrease was observed in the APOE level in the cingulate cortex in mice with PAE (Supplementary Fig. 3). The reduced activity of the cingulate cortex and anxiety behavior in mice with PAE, therefore, may depend on reduced APOE level in brain regions other than the cingulate cortex. Lower levels of plasma APOE was found both in our mouse model of acute PAE (Fig. 1) and in children with PAE (Fig. 6), who were likely periodically exposed to alcohol throughout gestation. Lower levels of *Apoe* mRNA were also found in PBMCs of mice with PAE (Fig. 1). Our results also suggest that APOE levels in blood samples may serve as a biomarker for neurobehavioral deficits associated with PAE, regardless of acute or chronic exposure.

Brain specific *Apoe* conditional KO mice show a reduction in the number of synapses and dendrites and AMPA/NMDA ratio, and infusion of recombinant APOE into lateral ventricles or genetic restoration of *Apoe* improves the phenotypes [10, 106]. In the present study, we tested the effects of a single dose of daily treatment with APOE-RA for 10 days on PAE mice. Similar to the previous finding that administration of a small APOE mimetic peptide Ac-hE18A-NH_2_ increases endogenous APOE in the mouse brain [71], we observed an increase in endogenous APOE in the brain after 10 days of APOE-RA administration to PAE mice (Fig. 4a and Supplementary Fig. 5e). Thus, the enhancement of intrinsic mechanism may augment the effects of APOE-RA treatment in alleviating the phenotype in PAE mice. In this regard, a recent study has shown that APOE can translocate to the nucleus and bind to DNA to function as a transcription factor in human glioblastoma cells [107]. To our surprise, however, the increase of endogenous APOE expression is not through the transcriptional control that is compromised by PAE (Supplementary Fig. 9). How APOE-RA increases APOE at the posttranscriptional level awaits further study. Given malfunctioning metabolism in FASD [108] and the increase of endogeneous APOE expression by APOE-RA (Supplementary Fig. 9), we are not able to exclude a possibility that APOE-RA improves neurobehavioral problems of mice with PAE through improvement of whole body metabolism in addition to the mechanism in which APOE-RA directly binds to receptors in brain as discussed the detail below.

LRP1 is a member of the LDL receptor family [109] that is highly expressed in neurons [110]. Cortical neuron-specific conditional knockout of *Lrp1* results in severe loss of motor function (both learning and locomotor) without histologically detectable structural abnormalities in the brain or effects on LTP and other major electrophysiological properties [12, 59]. A study has demonstrated that LRP1 is preferentially recognized by lipid-free recombinant APOE compared to other LDL receptor families [111]. Therefore, it is likely that the functions of APOE and APOE-RA in the neurobehavior that we observed are mediated by their binding to LRP1 [56]. Nevertheless, since APOE also binds to other LDL receptors including ApoER2 and VLDLR that also interact with PSD-95 at the postsynaptic terminals and play a role in synaptic plasticity [112], the relative contribution of each receptor to these functions of APOE and APOE-RA needs to be formally tested.

Our electrophysiological recording revealed that the sNMDA EPSC decay time in the motor cortex of PAE mice was significantly shorter and was restored by APOE-RA treatment to the level comparable to that of control mice (Fig. 3k). Given that a shorter decay time of sNMDA EPSC is known to be associated with reduced neuronal plasticity [113], the change in the kinetic property of NMDAR may be associated with the motor learning abilities. As we also observed reduction of CREB phosphorylation, which occurs in the downstream of NMDAR signaling and mediates transcription of many genes [68, 69], in the motor cortex by PAE (Supplementary Fig. 8), aberrant transcription of downstream genes involved in neuronal plasticity may also be involved in neurobehavioral problems in PAE.

Previous studies have shown that the levels of GluN2A and GluN2B subunits are associated with NMDAR decay kinetics [114] and developmental plasticity [115, 116], and the change in *Grin2b* to *Grin2a* expression ratio underlies the developmental shift of NMDAR decay time from slow to fast [70]. Indeed, overexpression of GluN2A has been shown to cause faster decay time than normal in cerebellar granule cells without affecting the amplitude of NMDA EPSC [117] similar to what we observed in our PAE mice (Fig. 3i, k). Enhanced GluN2A expression has also been associated with impaired long term synaptic plasticity, and learning and memory in mice [118, 119]. However, we did not observe significant difference in the expression of *Grin2a* and *Grin2b* in the motor cortex between control and PAE mice (Supplementary Fig. 9e–j). It is possible that the GluN2A to GluN2B ratio changes at the post-transcriptional level. Alternatively, but not mutually exclusively, since LRP1 has been shown to control the surface distribution and internalization of GluN2B in cortical neurons [120], intracellular trafficking and recycling of these NMDAR subunits may be affected by PAE and likewise by APOE-RA administration. In connection with this, chronic voluntary drinking during pregnancy has been shown to decrease synaptic GluN2B in the dentate gyrus in mouse offspring [121], while in another study, increase of GluN2B by PAE using similar regimen has been reported in the insular cortex of rat offspring [122]. The difference in these two studies may be due to the differences in the brain regions observed and/or alcohol dosages used: 5% [121] vs. 10% [122]. Further studies are needed to address whether the synaptic GluN2A and GluN2B protein levels are altered in cortical neurons by PAE.

Upregulation of KCNN2, which leads to increased medium afterhyperpolarization in cortical pyramidal neurons and directly contributes to motor learning deficits in PAE mice [13] is suppressed by APOE-RA treatment (Fig. 3c–f and Supplementary Fig. 5g). KCNN2 is degraded via the ubiquitin-proteasome pathway [66], which depends on NMDAR-mediated activation of calcium/calmodulin-dependent protein kinase II (CaMKII) in synaptic plasticity [123], while the effect of KCNN2 on CaMKII localization is required to refine synaptic plasticity [124]. Therefore, dysregulation of this mutual interactions of KCNN2 and NMDAR pathways due to APOE reduction may also be one of the mechanisms contributing to the motor learning deficits in PAE mice.

In conclusion, our study identified a mechanism by which PAE causes a decrease in the APOE level in the brain through an epigenetic mechanism, leading to neurobehavioral deficits. Although the number of patient samples is still small, the discovery of a PAE-susceptible SNP in the APOE enhancer and the finding of reduced plasma APOE levels, associated with lower cognitive performance in PAE individuals provide evidence for a genetic predisposition that interacts with PAE for the first time, and strongly support clinical translatability of APOE-targeted treatments for FASD.

## Supplementary information

Supplementary Figures

Supplementary Tables

## Data Availability

RNA and ATAC sequencing data are available as GSE202254 and GSE231517 at GEO, respectively.
