# Supplementary material for: Reduction of APOE accounts for neurobehavioral deficits in fetal alcohol spectrum disorders
Source: Mol Psychiatry. 2024 May 11;29(11):3364–80. doi: 10.1038/s41380-024-02586-6 (PMC11541007; doi:10.1038/s41380-024-02586-6)
Supplement: Supplementary file 1 — Supplementary Figures [file 41380_2024_2586_MOESM1_ESM.pdf]

Reduction of APOE accounts for neurobehavioral deficits in Fetal Alcohol Spectrum Disorders

SUPPLEMENTARY FIGURES

Hye M Hwang[1,#], Satoshi Yamashita[1,#], Yu Matsumoto[1], Mariko Ito[1], Alex Edwards[1], Junko Sasaki[1], Dipankar Dutta[1], Shahid Mohammad[1], Chiho Yamashita[1], Leah Wetherill[2], Tae-Hwi L Schwantes-An[2], Marco Abreu[2], Amanda H Mahnke[3], Sarah N Mattson[4], Tatiana Foroud[2], Rajesh C Miranda[3], Christina Chambers[5], Masaaki Torii[1,6,*], Kazue Hashimoto-Torii[1,6,*]

[1]Center for Neuroscience Research, The Children's Research Institute, Children's National Hospital, Washington, DC, USA

[2]Department of Medical and Molecular Genetics, Indiana University School of Medicine, Indianapolis, IN, USA

[3]Department of Neuroscience and Experimental Therapeutics, Texas A&M University School of Medicine, Bryan, TX, USA

[4]Center for Behavioral Teratology, San Diego State University, San Diego, CA, USA

[5]Department of Pediatrics, University of California San Diego, San Diego, CA, USA

[6]Departments of Pediatrics, and Pharmacology & Physiology, School of Medicine and Health Sciences, The George Washington University, Washington, DC, USA

[#] Equal contribution

*Correspondence

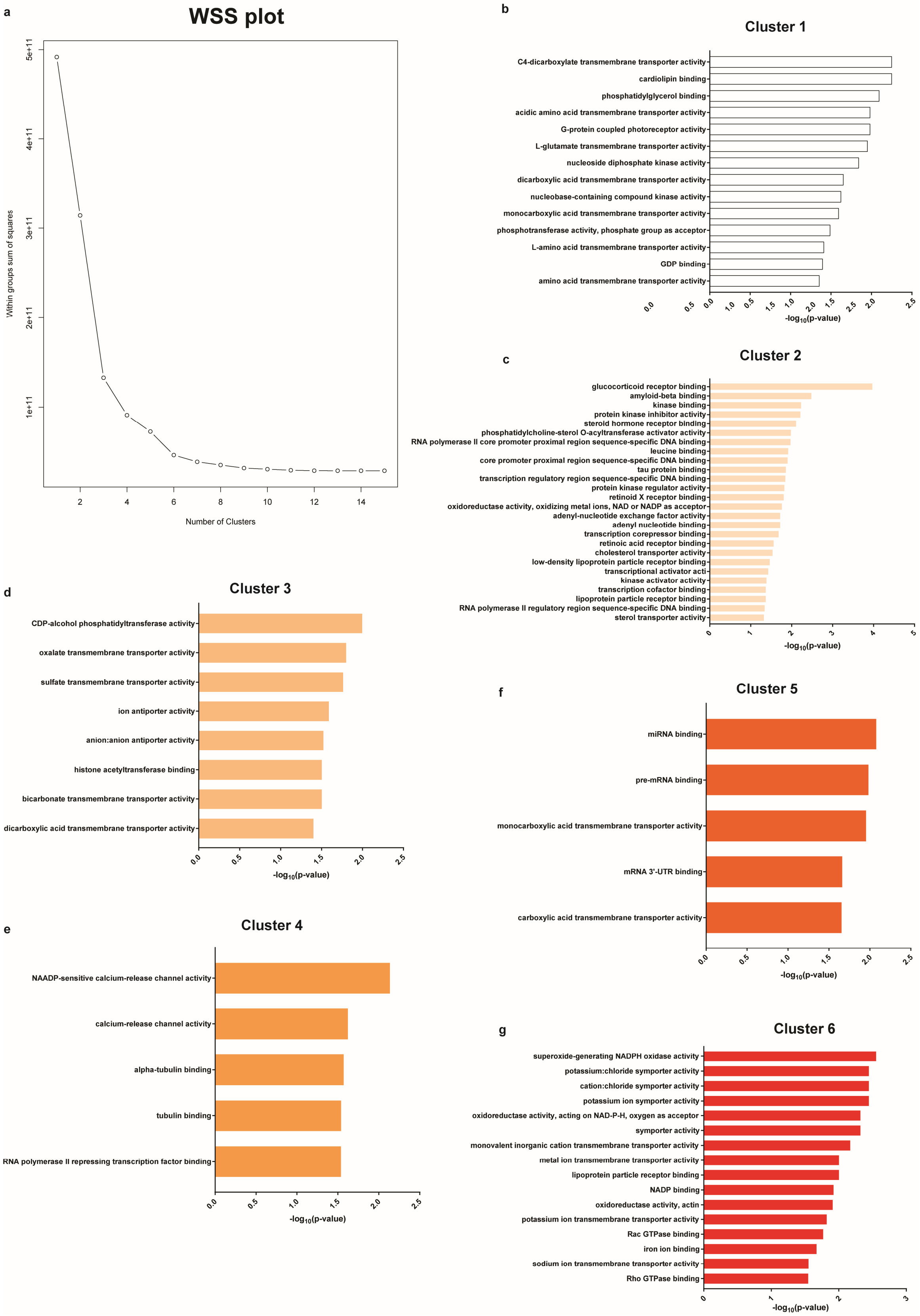

Supplementary Figure 1. Determination of the optimal cluster number for K-means clustering of B-cell DEGs and GOs enriched in each cluster.

a Within sum of squares (WSS) plot suggests the optimal number of K-means clustering for DEGs in B-cells to be six. **b-g** GOs enriched in indicated clusters are determined by Enrichr. Only statistically significant GOs are shown in the figures.

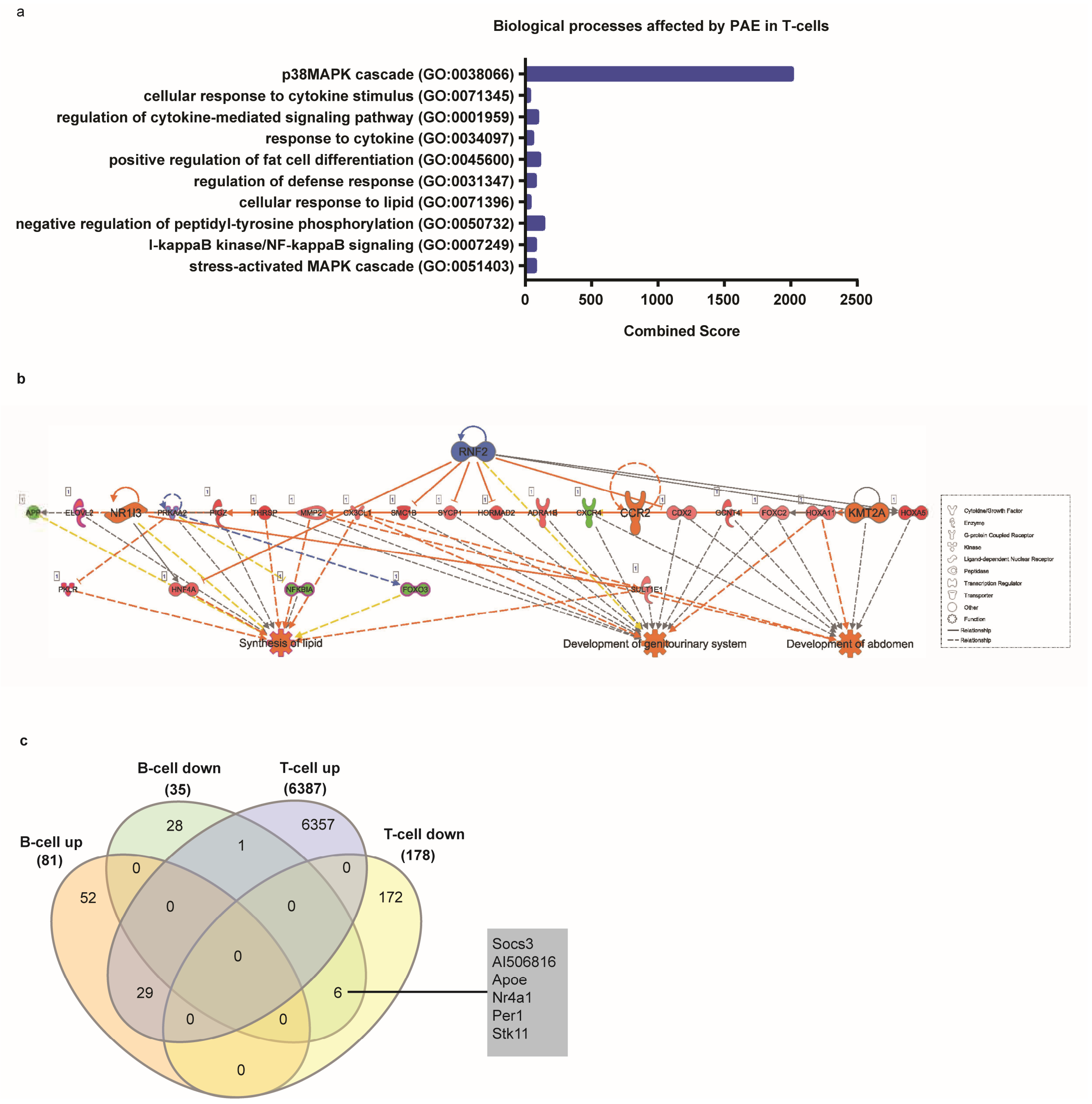

Supplementary Figure 2. T-cell RNA profiling reveals the impact of PAE on lipid-related pathways. **a** GOs enriched in the genes downregulated in T-cells are identified by Enrichr. No GOs are significantly enriched in the upregulated genes (data not shown). **b** The top regulator effect networks overlaid with diseases and disorders demonstrate that the activity of lipid synthesis is expected to be increased in T-cells by PAE. **c** There are 116 and 6565 differentially expressed genes in B-cells and T-cells, respectively, in PAE mice compared to the control mice. There are 36 overlapping genes, and *Apoe* is one of them. Venn-digram of the differentially expressed genes from B-cells and T-cells with a list of shared genes are shown.

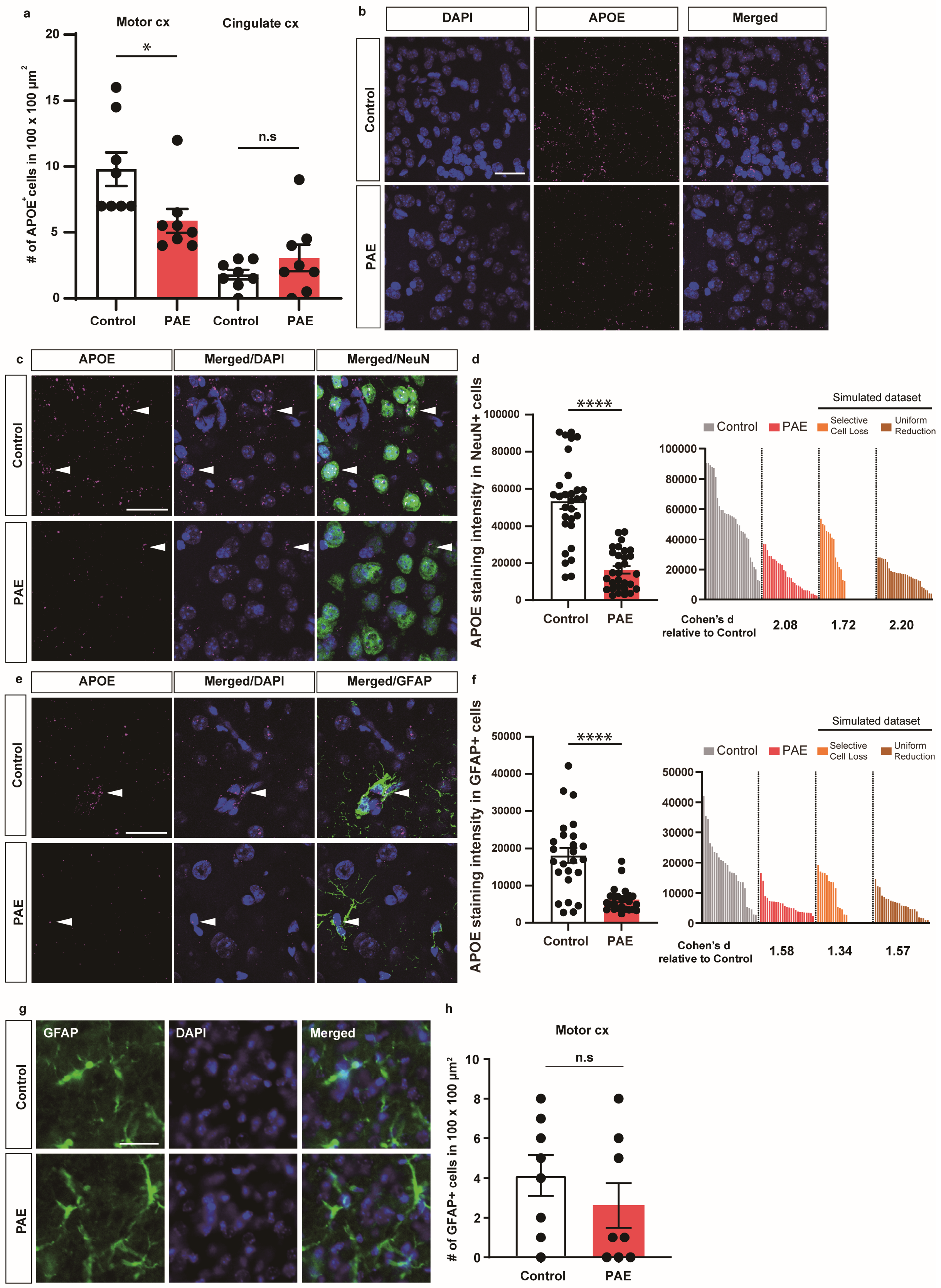

Supplementary Figure 3. APOE is decreased in the motor cortex.

a The number of APOE expressing cells in the motor cortex is reduced in PAE mice compared to control mice at P30. No significant difference was observed in the cingulate cortex. Control: n = 8; PAE: n = 8. **P*

= 0.026, n.s = not significant by two-tailed Student's t-test. Data represent mean ± s.e.m. **b** Representative images of APOE (magenta) and DAPI (blue) staining in layer V/VI of the motor cortex in control and PAE mice. Scale bar = 30 µm. **c** Representative images of NeuN (green), APOE (magenta), and DAPI (blue) staining in layer V/VI of the motor cortex in control and PAE mice at P30. Arrowheads point at the APOE expressing cells. Scale bar = 30 µm. **d** Graphs show quantification of APOE staining intensity in each NeuN-positive cells (n = 30 from 3 animals per group), indicating a reduction of APOE immunolabeling in the cortex of PAE mice. Each dot in the bar graph (left) and each bar in the histogram (right) represents an individual cell. Creation of simulated datasets was found in Material and Methods. Cohen's d was calculated to determine the effect size based on the difference between the means of data from control mice and the other datasets (PAE mice and two simulation models). ****P < 0.0001 by two-tailed Student's t-test. Data represent mean ± s.e.m. **e** Representative images of GFAP (green), APOE (magenta), and DAPI (blue) staining in layer V/VI of the motor cortex in control and PAE mice at P30. Arrowheads point at the APOE expressing cells. Scale bar = 30 µm. **f** Graphs show quantification of APOE staining intensity in each GFAP-positive cells (n = 25 from 3 animals per group), indicating a reduction of APOE immunostaining intensity in the cortex of PAE mice. Each dot in the bar graph and each bar in the histogram represents an individual cell. Creation of simulated datasets was found in Material and Methods. Cohen's d was calculated to evaluate the effect size based on the difference between the means of data from control mice and the other datasets (PAE mice and two simulation models). ****P < 0.0001 by two-tailed Student's t-test. Data represent mean ± s.e.m. **g** Representative images of GFAP (green) and DAPI (blue) staining in layer V/VI of the motor cortex in control and PAE mice at P30. Scale bar = 50 µm. **h** Quantification reveals no significant difference in the number of GFAP-positive cells in the motor cortex between control and PAE mice at P30 (n = 8 per group). n.s = not significant by two-tailed Student's t-test. Data represent mean ± s.e.m.

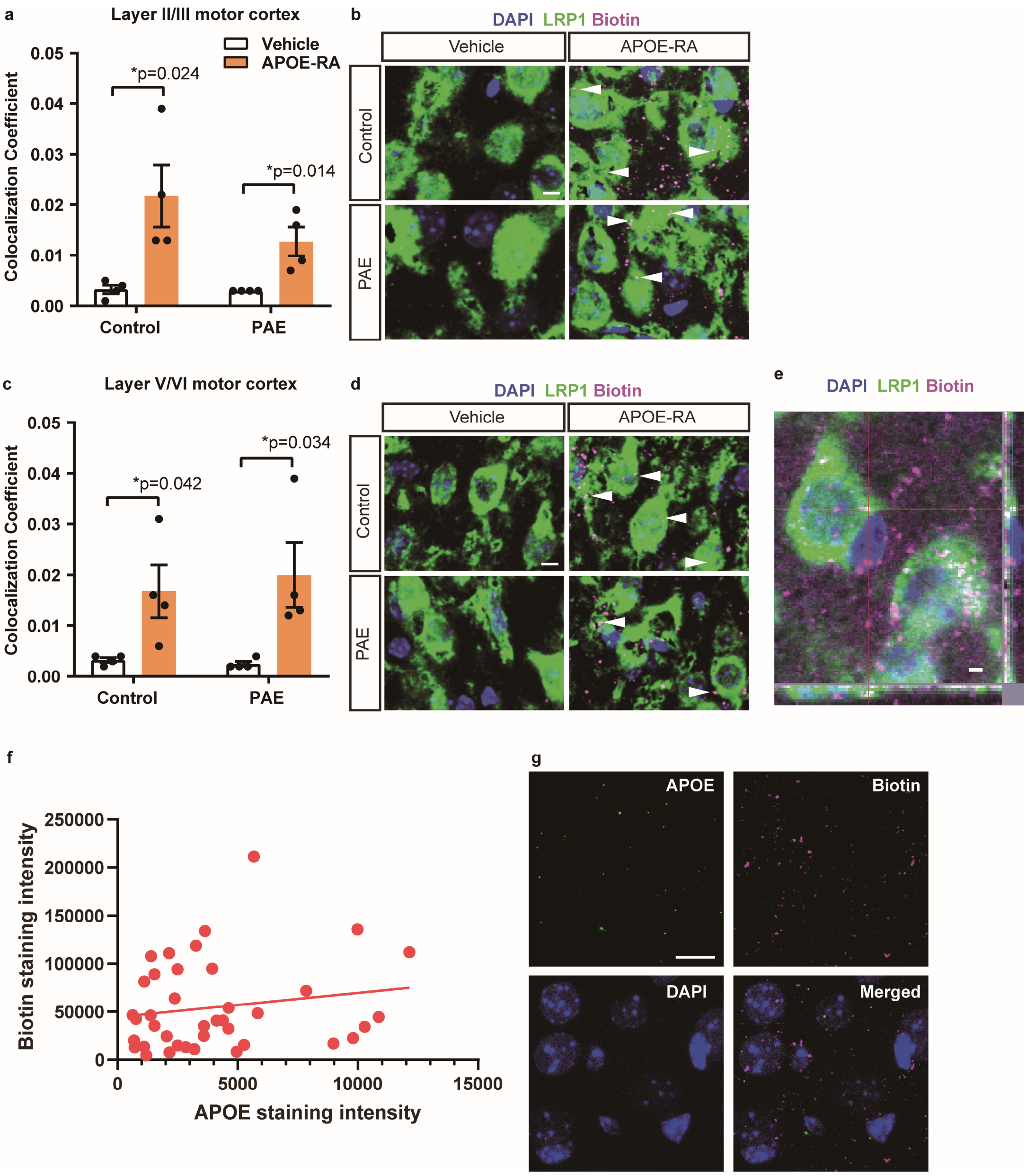

Supplementary Figure 4. APOE-RA administered by intraperitoneal injection reaches the brain. **a**, **c** Graphs show colocalization of biotin and LRP1 staining in the motor cortex of control and PAE mice that were administered biotinylated APOE-RA intraperitoneally. Vehicle only was administered as the control (n = 4 per group). Brains were collected 5 minutes post administration at P20. **P* < 0.05 by two-tailed Student's t-test. Data represent mean ± s.e.m. **b**, **d** Representative images of biotin (magenta), LRP1 (green), and DAPI (blue) staining. Arrowheads pointing at the biotin staining in LRP1-positive cell. Scale bar = 5 µm. **e** A higher magnification image of biotin and LRP1 double labeling with orthogonal projection. Scale bar = 5 µm. **f** Pearson's correlation analysis reveals no significant correlation (R^2 = 0.031, P = 0.287) between the intensities of biotinylated APOE-RA detection and immunolabeling of endogeneous APOE in each cell in the motor cortex of PAE mice at P20. Brains were fixed 5 minutes after the last injection of biotinylated APOE-RA. Each dot represents an individual cell (n = 39 from 3 animals). **g** Representative images of APOE (green), biotin (magenta), and DAPI (blue) staining. Scale bar = 10 µm.

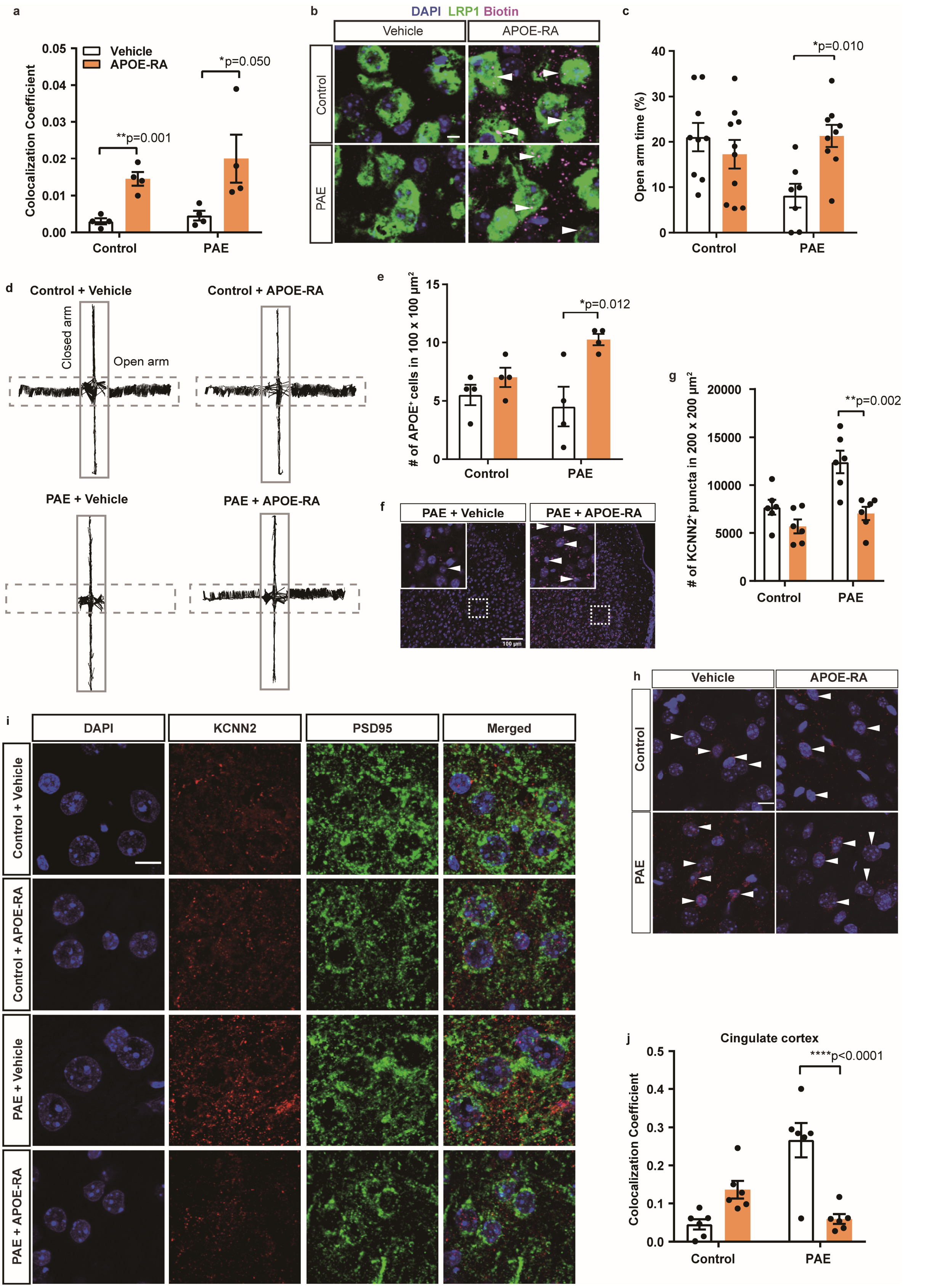

Supplementary Figure 5. Postnatal APOE-RA treatment mitigates anxiety behavior of PAE mice.
a Significant colocalization of biotin and LRP1 labeling is found in the cingulate cortex of control and PAE mice 5 minutes after intraperitoneal administration of biotinylated APOE-RA at P20. Vehicle only was

administered as the control (n = 4 per group). *P < 0.05, **P < 0.01 by two-tailed Student's t-test. Data represent mean ± s.e.m. **b** Representative images of biotin (magenta), LRP1 (green), and DAPI (blue) staining. Arrowheads indicate the biotin labeling within LRP1-positive cells. Scale bar = 5 µm. **c** APOE-RA-treated PAE mice spend significantly more time in the open arm compared to vehicle-treated PAE mice (control+vehicle: n = 9; control+APOE-RA: n = 10; PAE+vehicle: n = 7; PAE+APOE-RA: n = 9). EPM tests were done around P30. *P < 0.05 by two-way ANOVA with simple main effect test. Data represent mean ± s.e.m. **d** Representative images of line tracks in the EPM test. A dashed box outlines an open arm, and a solid box outlines a closed arm. **e** APOE-RA treatment increases the number of cells positive for endogenous APOE expression in the cingulate cortex in PAE mice (n = 4 per group). *P < 0.05 by two-way ANOVA with Bonferroni's multiple comparison test. Data represent mean ± s.e.m. **f** Representative images of APOE immunolabeling (magenta) in the cingulate cortex in the PAE mice treated with vehicle or APOE-RA. Cell nuclei were labeled with DAPI (blue). Dotted boxes indicate the region shown in the inset at higher magnification. Arrowheads indicate the APOE-positive cells. Scale bar = 100 µm. **g** The number of KCNN2 puncta is significantly decreased in the cingulate cortex of PAE mice after the APOE-RA treatment compared to vehicle treatment (n = 6 per group). **P < 0.01 by two-way ANOVA with Bonferroni's post hoc test. Data represent mean ± s.e.m. **h** Representative images of KCNN2 (red) and DAPI (blue) staining. The white arrowheads point at the KCNN2 punctae-containing cells. Scale bar = 10 µm. **i** Representative images of KCNN2 (red), PSD95 (green), and DAPI (blue) staining in the cingulate cortex of indicated groups of mice. Scale bar = 10 µm. **j** The colocalization of KCNN2 and PSD95 is significantly reduced in the cingulate cortex of PAE mice after the APOE-RA treatment for 10 days (n = 6 per group). ****P < 0.0001 by two-way ANOVA with simple main effect test. Data represent mean ± s.e.m.

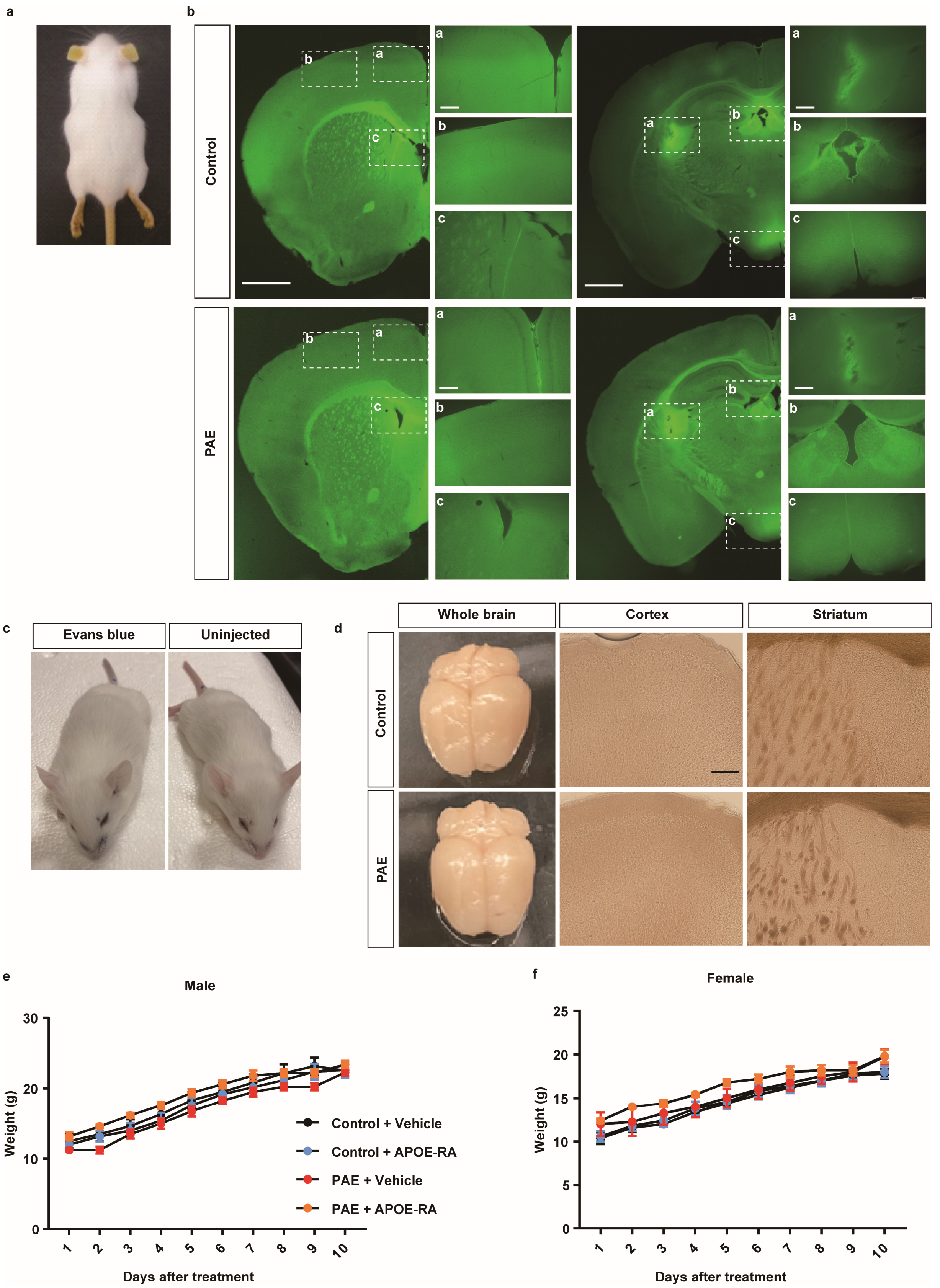

Supplementary Figure 6. Blood-brain barrier (BBB) is intact in the brains of PAE mice, and APOE-RA treatment does not affect the body weight.

a Image of a P30 mouse 30 minutes post injection of sodium fluorescein. **b** Representative images of brain slices show no changes in BBB permeability to sodium fluorescein in PAE mice compared to control mice (n = 6 per group). Scale bar = 1 mm. **c** Images of P30 mice 30 minutes post injection of Evans blue dye (left) and without injection (right). **d** Control and PAE mice injected with Evans blue dye do not show any difference in BBB permeability to the dye (n = 2 per group). Scale bar = 200 µm. **e**, **f** There is no statistically significant difference in the body weight between control and PAE groups regardless of the treatment type (vehicle or APOE-RA) (Male: control+vehicle: n = 4, control+APOE-RA: n = 5, PAE+vehicle: n = 4, PAE+APOE-RA: n = 5; Female: control+vehicle: n = 5, control+APOE-RA: n = 5, PAE+vehicle: n = 3, PAE+APOE-RA: n = 5). Two-way repeated measures ANOVA with post hoc simple main effect test (**e**) or Tukey's test (**f**). Data represent mean ± s.e.m.

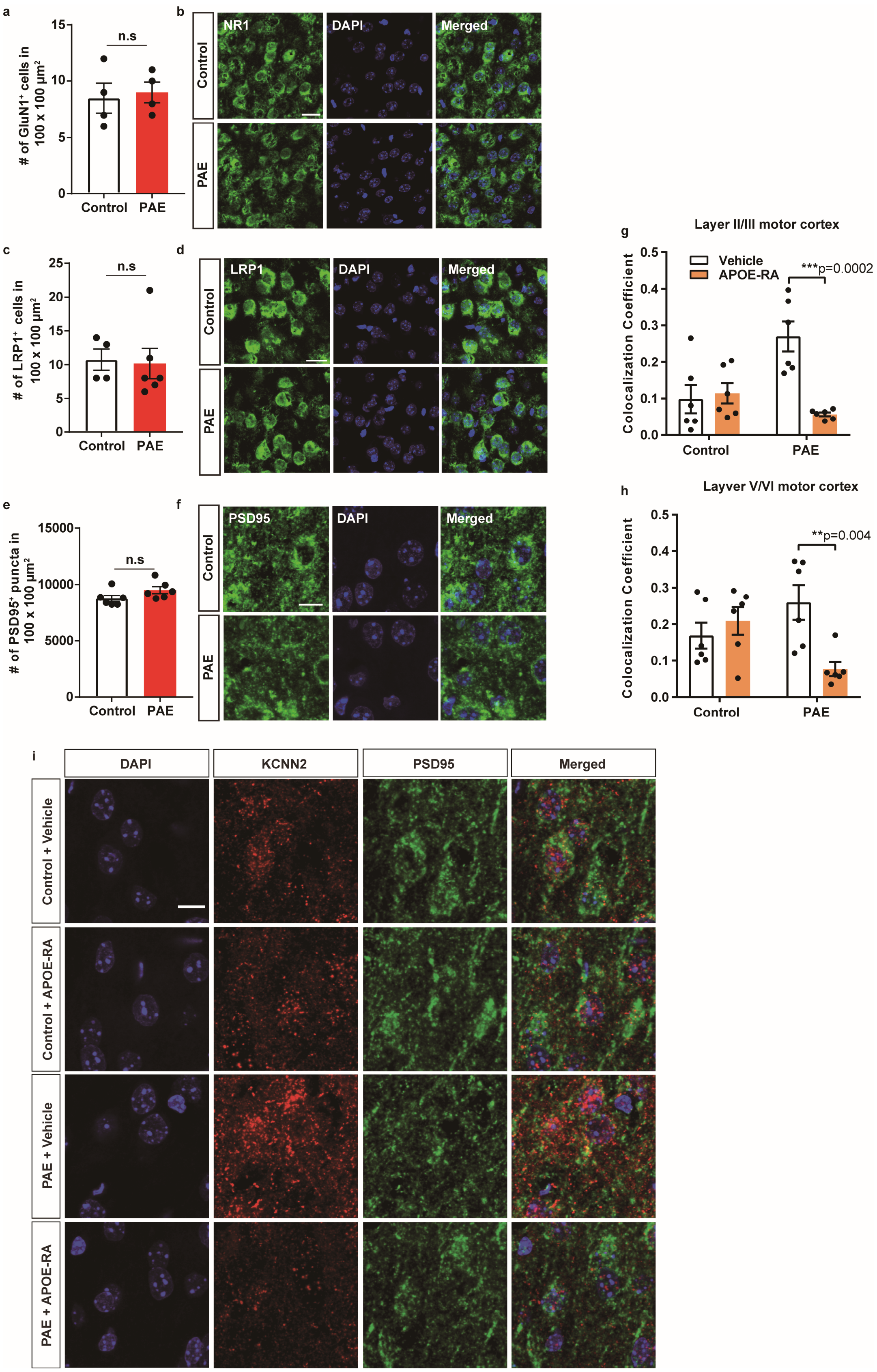

Supplementary Figure 7. PAE does not affect the expression level of GluN1, LRP1, and PSD95 in layer V/VI of the mouse motor cortex at P30.

a, **c**, **e** The number of GluN1 (**a**) and LRP1 (**c**) -positive cells and the PSD95 (**e**) puncta are not changed in PAE mice compared to control mice [n = 4 per group (**a**); control: n = 4, PAE: n = 6 (**c**), or n = 6 per group (**e**)]. n.s = not significant by two-tailed Student's t-test. Data represent mean ± s.e.m. **b**, **d**, **f** Representative images of GluN1 (**b**), LRP1 (**d**), and PSD95 (**f**) immunohistochemistry (green), respectively. Nuclei were labeled with DAPI (blue). Scale bars = 20 µm (**b, d**) and 10 µm (**f**). **g**, **h** The colocalization of KCNN2 and PSD95 is significantly reduced in the motor cortex of PAE mice after the APOE-RA treatment (n = 6 per group). $^{**}P < 0.01$, $^{***}P < 0.001$ by two-way ANOVA with simple main effect test. Data represent mean ± s.e.m. **i** Representative images of KCNN2 (red), PSD95 (green), and DAPI (blue) staining in layer V/VI of the motor cortex in indicated groups. Scale bar = 10 µm.

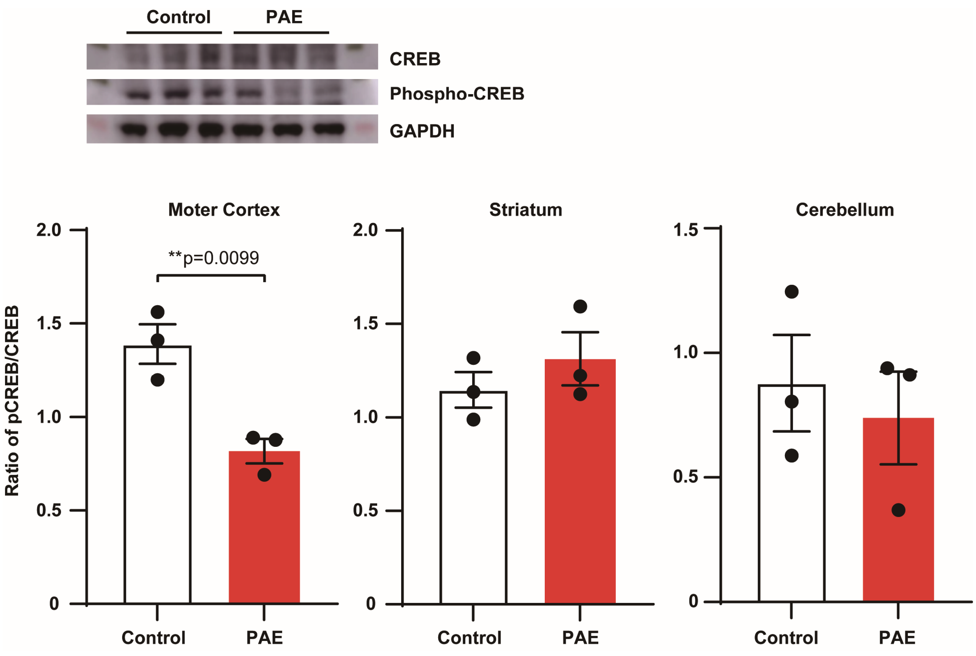

Supplementary Figure 8. Phosphorylation of CREB is reduced in the motor cortex but not in the striatum and cerebellum in PAE mice at P30.

Images show the bands of pan-CREB (top), phospho-CREB (middle) and GAPDH (bottom) extracted from the motor cortex of the indicated animal groups. The side lanes are for the molecular weight marker. Graphs show the ratio of the signal intensity between phospho-CREB and pan-CREB [n = 3 samples each (two males and one female from different litters)]. Unpaired t-test. Data represent mean ± s.e.m.

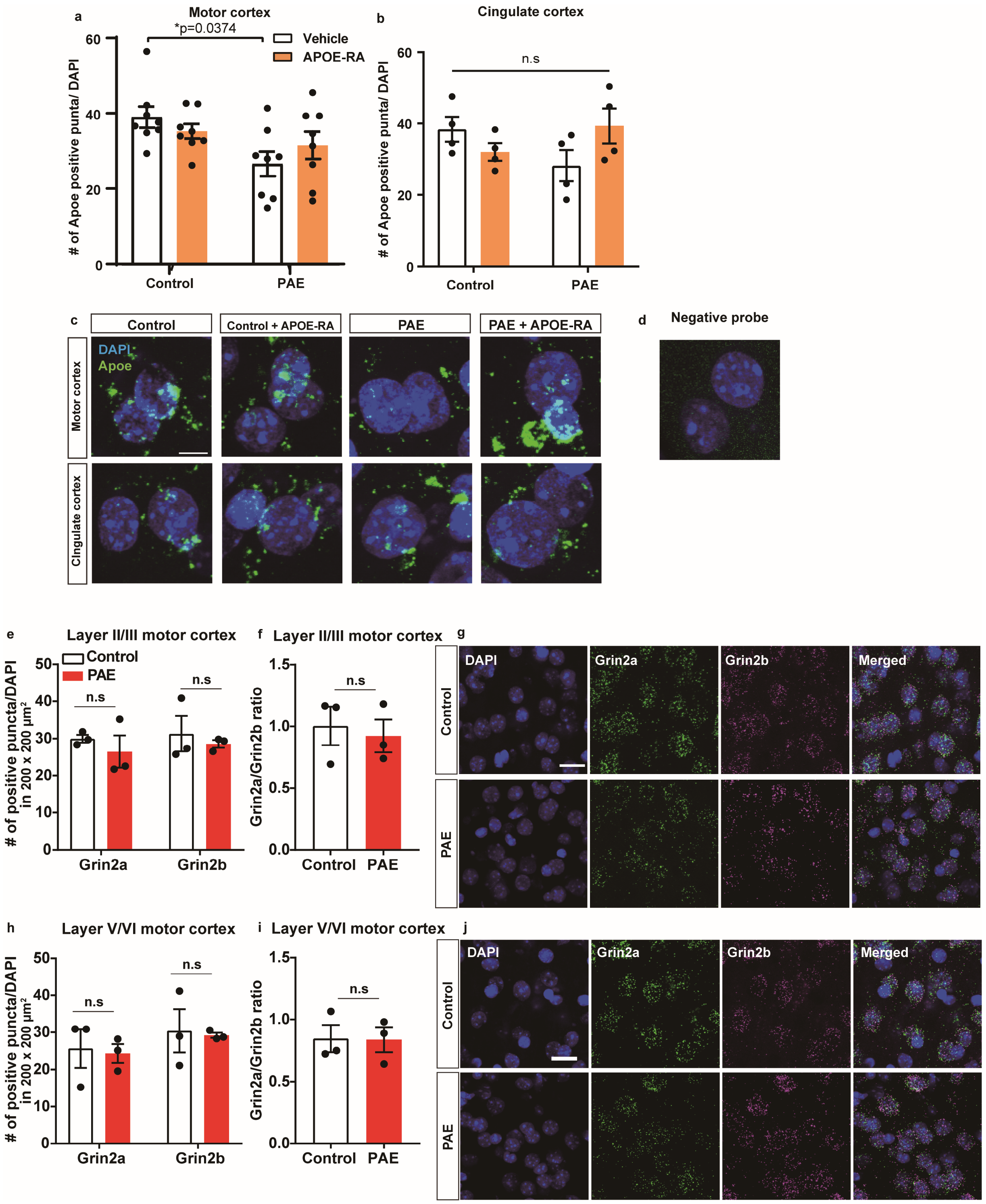

Supplementary Figure 9. PAE decreases *Apoe* mRNA expression level in the motor cortex without changes in *Grin2a* and *Grin2b* expression levels.

a The motor cortex in PAE mice shows a significant reduction in *Apoe* mRNA expression compared to control mice at P30. Postnatal treatment with APOE-RA does not significantly alter the expression (n = 8 per group). *$P < 0.05$ by two-way ANOVA followed by Tukey's post hoc test. Data represent mean ± s.e.m. **b** There is no significant difference in the *Apoe* mRNA expression level between indicated groups in the cingulate cortex (n = 4 per group). n.s = not significant by two-way ANOVA followed by Tukey's post hoc test. Data represent mean ± s.e.m. **c** Representative RNAscope images probed with *Apoe* (green) with DAPI labeling (blue) in the motor and cingulate cotices of the indicated groups. Scale bar = 5 µm. **d** Representative

RNAscope image stained with a negative control probe (green) with DAPI labeling (blue) in the motor cortex. **e**, **f** There are no changes in the *Grin2a* and *Grin2b* expression and their ratio in layer II/III of the motor cortex between control and PAE mice (n = 3 per group). n.s = not significant by two-tailed Student's t-test. Data represent mean ± s.e.m. **g** Representative RNAscope images probed with *Grin2a* (green) and *Grin2b* (blue) in layer II/III of the motor cortex. Scale bar = 20 µm. **h**, **i** There are no changes in the *Grin2a* and *Grin2b* expression or their ratio in layer V/VI of the motor cortex between control and PAE mice (n = 3 per group). n.s = not significant by two-tailed Student's t-test. Data represent mean ± s.e.m. **j** Representative RNAscope images probed with *Grin2a* (green) and *Grin2b* (blue) with DAPI labeling (blue) in layer V/VI of the motor cortex. Scale bar = 20 µm.
